# Two vs three cycles of neoadjuvant sintilimab plus chemotherapy for resectable non-small-cell lung cancer: neoSCORE trial

**DOI:** 10.1038/s41392-023-01355-1

**Published:** 2023-04-10

**Authors:** Miner Shao, Jie Yao, Yunke Wang, Lufeng Zhao, Baizhou Li, Lili Li, Zuqun Wu, Zexin Chen, Junqiang Fan, Fuming Qiu

**Affiliations:** 1 Department of Medical Oncology, The Second Affiliated Hospital, Zhejiang University School of Medicine, Hangzhou, Zhejiang China; 2 Cancer Institute, Key Laboratory of Cancer Prevention and Intervention, Ministry of Education, The Second Affiliated Hospital, Zhejiang University School of Medicine, Hangzhou, Zhejiang China; 3 Key Laboratory of Tumor Microenvironment and Immune Therapy of Zhejiang Province, The Second Affiliated Hospital, Zhejiang University School of Medicine, Hangzhou, Zhejiang China; 4 Department of Thoracic Surgery, The Second Affiliated Hospital, Zhejiang University School of Medicine, Hangzhou, Zhejiang China; 5 Department of Pathology, The Second Affiliated Hospital, Zhejiang University School of Medicine, Hangzhou, Zhejiang China; 6 Department of Respiratory Medicine, The Second Affiliated Hospital, Zhejiang University School of Medicine, Hangzhou, Zhejiang China; 7 Department of Biostatistics, The Second Affiliated Hospital, Zhejiang University School of Medicine, Hangzhou, Zhejiang China; 8 Cancer Center, Zhejiang University, Hangzhou, Zhejiang China

**Keywords:** Lung cancer, Tumour immunology

**Dear Editor**,

Lung cancer is the predominant cause of cancer-related deaths globally, with non-small cell lung cancer (NSCLC) accounting for 85% of all cases.^1^ The five-year overall survival (OS) after surgery remains poor because of the high rate of postoperative recurrence and metastasis. Recently, immune checkpoint inhibitors (ICIs) have shown promising efficacy in resectable NSCLC. A previous study reported that two cycles of neoadjuvant sintilimab achieved a major pathological response (MPR) rate of 40.5% in stage IA-IIIB NSCLC.^2^ Recently, the CheckMate-816 trial demonstrated that neoadjuvant nivolumab plus chemotherapy produced superior pathological complete response (pCR) rates compared to chemotherapy alone.^3^ At present, no prospective randomized study evaluating different cycles of neoadjuvant immuno-chemotherapy has been conducted. On the basis of the inspiring efficacy and the important gap of optimal treatment period in neoadjuvant ICI plus chemotherapy, we conducted this study to evaluate the outcome of two vs three cycles of neoadjuvant sintilimab with platinum-based chemotherapy in resectable IB-IIIA NSCLC patients (NCT04459611).

Between July 2020 and September 2021, we screened 64 patients, of whom 60 eligible patients were randomized and received either two (*n* = 29) or three (*n* = 31; Fig. 1a) cycles of neoadjuvant treatment. The demographic characteristics were well balanced between both arms (Supplementary Table 1). Among the 60 randomized patients, 91.7% (55/60) underwent surgery. Two patients (6.9%) in three-cycle arm underwent pneumonectomy. Detailed data are presented in Supplementary Table 2–4.

**Fig. 1:**
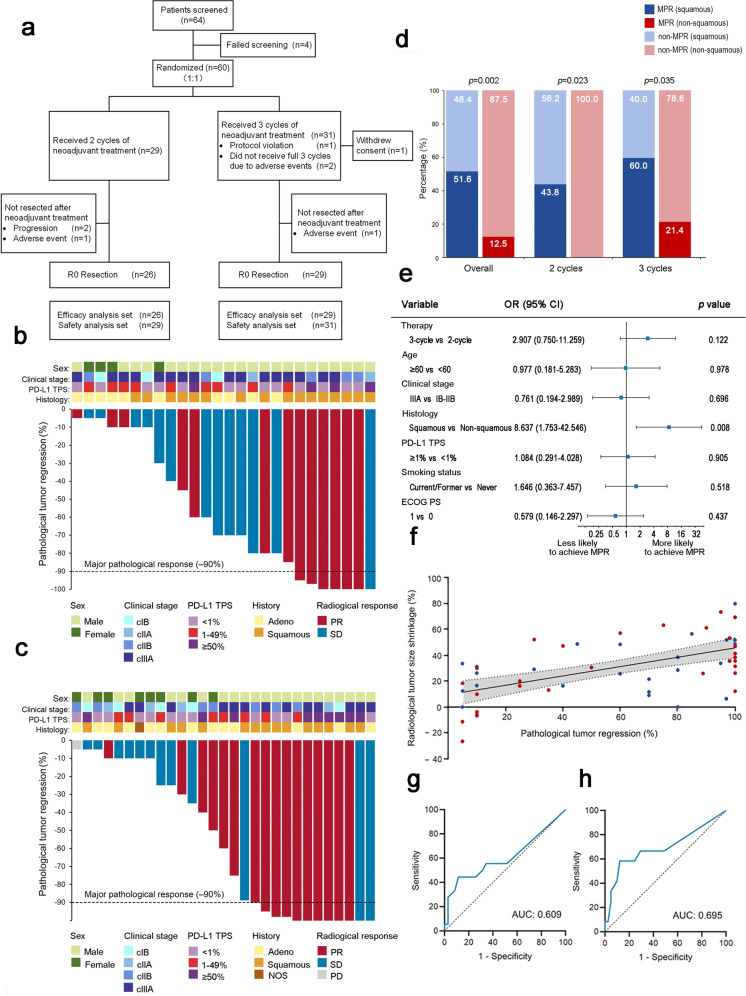
**a** CONSORT Diagram. **b**, **c** Waterfall plot. The percentage of pathological regression in the resected primary tumor after (**b**) two cycles or (**c**) three cycles of neoadjuvant sintilimab plus chemotherapy. The dashed lines represent major pathological response (–90%). **d** The proportion of patients with different responses to neoadjuvant immuno-chemotherapy. **e** Multivariate logistic regression for major pathological response. **f** Correlation between pathological response and pathological response (Spearman’s r = 0.55, *p* < 0.0001). The blue points represent the patients in two-cycle arm, and the red points represent the patients in three-cycle arm. The solid line represents the linear regression line, and the dashed lines represent the 95% confidence intervals. The receiver operating characteristic curves for (**g**) major pathological response and (**h**) pathological complete response. PD-L1 programmed cell death–ligand 1, TPS tumor proportion score, PR partial response, SD stable disease, NOS not otherwise specified, PD progressive disease, MPR major pathological response, OR odds ratio, AUC area under the curve

Of the 55 patients who underwent surgery, 19 (34.5%; 95% CI: 22.2–48.6%) patients achieved MPR (Supplementary Table 5). The MPR rate was 26.9% (7/26, 95% CI: 11.6–47.8%) in the two-cycle arm vs 41.4% (12/29, 95% CI: 23.5–61.1%; *p* = 0.260; Fig. 1b, c) in the three-cycle arm. In a planned subgroup analysis of MPR according to baseline characteristics, no significant differences were observed between subgroups (Supplementary Fig. 1).

A superior MPR rate was observed in squamous NSCLC (51.6%, 16/31) than in non-squamous NSCLC (12.5%, 3/24; *p* = 0.002; Fig. 1d). Multivariate logistic regression confirmed that the squamous subtype was significantly related to MPR (odds ratio=8.637, 95% CI: 1.753-42.546, *p* = 0.008; Fig. 1e). To determine the cause of low MPR rate in non-squamous NSCLC, 95.8% (23/24) of non-squamous NSCLC patients having sufficient tumor tissue underwent driver mutation next-generation sequencing (NGS) testing. Driver mutations were detected in 21 patients, including EGFR (13/23, 56.5%), ERBB2 (2/23, 8.7%), MET (2/23, 8.7%), KRAS-G12C (2/23, 8.7%), ALK (1/23, 4.3%), and RET (1/23, 4.3%). Of the two patients who achieved MPR, one had an EGFR mutation, and the other had a KRAS-G12C mutation (Supplementary Table 6).

The pCR rate was 19.2% (5/26; 95% CI: 6.6–39.4%) in the two-cycle arm, and 24.1% (7/29; 95% CI: 10.3–43.5%; *p* = 0.660) in the three-cycle arm (Supplementary Table 7). The objective response rates (ORRs) were 50.0% (13/26; 95% CI: 29.9–70.1%) and 55.2% (16/29; 95% CI: 35.7–73.6%; *p* = 0.701), respectively (Supplementary Fig. 2). A significant association was observed between radiological and pathological responses (Spearman’s r = 0.55, *p* < 0.0001; Fig. 1f).

At the time of data cutoff (September 30, 2022), the median follow-up was 20.4 months (95% CI: 19.8–20.9) from first day of treatment. The median OS and disease-free survival (DFS) were not reached in patients received surgery (Supplementary Fig. 3). The 12-month OS rates were 92.3% in the two-cycle arm and 86.2% in the three-cycle arm. The 12-month DFS rates were 84.4% and 82.8%, respectively. Kaplan‒Meier curves of patients with MPR and non-MPR were shown in Supplementary Fig. 4.

We assessed the predictive value of programmed cell death ligand-1 tumor proportion score (PD-L1 TPS) for MPR and pCR. The area under the curves (AUCs) were 0.609 and 0.695 for predicting the MPR and pCR rates, respectively. The optimal PD-L1 TPS cutoff value was 45% for predicting MPR (sensitivity, 44.4%; specificity, 88.6%; *p* = 0.198) and pCR (sensitivity, 58.3%; specificity, 87.8%; *p* = 0.041; Fig. 1g, h).

Among the 55 patients who underwent surgery, 23.1% (6/26) in the two-cycle arm and 31.0% (9/29) in the three-cycle arm developed postoperative complications, most of which were grades I-II. Treatment-related adverse events (TRAEs) were summarized in Supplementary Table 8. Among the 60 randomized patients, 31.0% (9/29) of patients in the two-cycle arm and 29.0% (9/31) of patients in the three-cycle arm had grade ≥3 TRAEs. Grade ≥3 immune-related AEs included colitis in one and pneumonia in two patients. TRAEs during adjuvant therapy were summarized in Supplementary Table 9.

To our knowledge, this is the first randomized trial to assess the efficacy and safety of different cycles of immuno-chemotherapy for resectable NSCLC in the neoadjuvant setting. Two to four cycles of neoadjuvant immuno-chemotherapy are generally used in most clinical trials. According to a phase 2 study using four cycles of neoadjuvant atezolizumab plus chemotherapy, 17% of patients do not receive four full cycles of treatment due to toxicity and proceed to surgery early.^4^ Out of concern for toxicity, we perform this study to compare two and three cycles of neoadjuvant immune-chemotherapy. Three cycles of treatment present a 14.5% increase in MPR rate in comparison to two cycles. The difference does not achieve statistical significance (*p* = 0.260) possibly because of the small sample size. Moreover, the addition of only one dose of treatment may not be adequate to reach significant differences in pathological regression. Although two patients in three-cycle arm underwent pneumonectomy, they recovered well after surgery with no major postoperative complications. Overall, the toxicities were predictable and manageable in both arms.

Notably, squamous NSCLC achieve more favorable pathological remissions to neoadjuvant immuno-chemotherapy than non-squamous NSCLC. In contrast, the NADIM and CheckMate-816 trials show consistent benefits in squamous and non-squamous NSCLC.^3,5^ Driver mutation NGS testing shows a high mutation rate in non-squamous NSCLC, which provides a possible explanation for the low MPR rate in non-squamous subtype. Additionally, Gao et al. report that among EGFR-negative patients, squamous NSCLC has superior MPR rates compared to non-squamous NSCLC in a single-agent neoadjuvant sintilimab setting.^2^ Further studies are warranted to clarify the immune microenvironment differences between squamous and non-squamous NSCLC.

We have found a significant association between the PD-L1 TPS and pathological responses. Nonetheless, PD-L1 TPS is not sufficiently sensitive to predict pathological response. Five cases in three-cycle group have enlarged tumor on imaging and are found to have a high proportion of residual tumor cells after surgery (Supplementary Table 10). For patients insensitive to immuno-chemotherapy, prolonged therapy may increase the risk of disease progression. Further studies are required to identify better biomarkers for predicting treatment efficacy.

The PACIFIC trial reports that durvalumab monotherapy for one year lead to durable antitumor activity and a decreased rate of metastasis, indicating that prolonged PD-1/PD-L1 blockades treatment have contribution to sustained survival benefit. There are 14 patients in our study underwent maintenance therapy of sintilimab, we will further analyze and report these data. The limitations of this study are the inclusion of patients with EGFR/ALK mutations, and the limited sample size. Additionally, survival data are immature and follow-up is still ongoing.

In conclusion, our study provides insight into the treatment cycles of neoadjuvant immuno-chemotherapy. Increasing the number of cycles of neoadjuvant treatment from two to three leads to a numerical improvement in MPR with good tolerability. To further verify that prolonged neoadjuvant treatment leads to an improvement in MPR, we will conduct a phase 3 trial (NCT05429463) to compare three vs four cycles of neoadjuvant immuno-chemotherapy in resectable squamous NSCLC.

## Supplementary information

Supplementary Materials

Protocol

## Data Availability

All data are available by requirements to the corresponding authors F.M.Q. (qiufuming@zju.edu.cn) and J.Q.F. (zrxwk@zju.edu.cn).
