# Supplementary material for: Two vs three cycles of neoadjuvant sintilimab plus chemotherapy for resectable non-small-cell lung cancer: neoSCORE trial
Source: Signal Transduct Target Ther. 2023 Apr 10;8:146. doi: 10.1038/s41392-023-01355-1 (PMC10083171; doi:10.1038/s41392-023-01355-1)
Supplement: Supplementary file 1 — Supplementary Materials [file 41392_2023_1355_MOESM1_ESM.docx]

Supplementary Materials for

Two vs three cycles of neoadjuvant sintilimab plus chemotherapy for resectable non-small-cell lung cancer: neoSCORE trial

Miner Shao^1^; Jie Yao ^2^; Yunke Wang^1^; Lufeng Zhao^2^; Baizhou Li^3^; Lili Li^1^; Zuqun Wu^4^; Zexin Chen^5^; Junqiang Fan^2^; Fuming Qiu^1^.

1 Department of Medical Oncology, The Second Affiliated Hospital, Zhejiang University School of Medicine, Hangzhou, Zhejiang, China.

2 Department of Thoracic Surgery, The Second Affiliated Hospital, Zhejiang University School of Medicine, Hangzhou, Zhejiang, China.

3 Department of Pathology, The Second Affiliated Hospital, Zhejiang University School of Medicine, Hangzhou, Zhejiang, China.

4 Department of Respiratory Medicine, The Second Affiliated Hospital, Zhejiang University School of Medicine, Hangzhou, Zhejiang, China.

5 Department of Biostatistics, The Second Affiliated Hospital, Zhejiang University School of Medicine, Hangzhou, Zhejiang, China.

Correspondence to: [qiufuming@zju.edu.cn, Fuming Qiu](mailto:xxxxx@xxxx.xxx) or zrxwk@zju.edu.cn, Junqiang Fan

**This PDF file includes:**

Materials and Methods

Figures S1 to S4

Tables S1 to S10

Materials and Methods

**Patients**

NeoSCORE was a prospective, open-label, single-center, randomized phase 2 study that evaluated different cycles of neoadjuvant immuno-chemotherapy for resectable non-smallcell lung cancer (NSCLC). We enrolled patients (aged 18 to 75 years) with cytologically or histologically proven NSCLC. Other inclusion criteria included previously untreated stage cIB-IIIA NSCLC (American Joint Committee on Cancer [AJCC, 8th edition]^1,2^), at least one measurable lesion (Response Evaluation Criteria in Solid Tumors [RECIST, version 1.1]^3^), adequate organ function, life expectancy more than six months and Eastern Cooperative Oncology Group (ECOG) performance status of 0 or 1.^4^ Key exclusion criteria included previous checkpoint inhibitor therapy, a history of malignancy within the past five years, serious systemic diseases, and active autoimmune disease requiring systemic treatment. The detailed protocol was presented in the Supplementary Materials.

The trial was approved by the local independent ethics committee and conducted according to Good Clinical Practice and the Declaration of Helsinki. An independent data safety monitoring board (DSMB) monitored the study and reviewed the data of the prespecified interim analyses. All patients provided written informed consent before enrollment.

**Study design and Treatments**

In this trial, eligible patients were randomized to the two- or three-cycle arm in a 1:1 ratio. A stratified-block randomization design was used, with a block size of six, and the stratification factor was programmed cell death ligand-1 (PD-L1) expression (≥1% vs <1%). The patients received neoadjuvant intravenous sintilimab (200 mg; Innovent Biologics, Suzhou, China) plus chemotherapy (carboplatin area under the curve [AUC] 5 plus nab-paclitaxel 260mg/m^2^ for squamous NSCLC or pemetrexed 500mg/m^2^ for non-squamous NSCLC) every three weeks. Surgery was performed within the fourth week (± 7 days) from the last dose of neoadjuvant treatment. After operation, the patients received one or two cycles of the same regimen to reach four doses of perioperative therapy in total. Radiotherapy was administered at the discretion of the investigators based on the patient’s clinical situation and pathological staging. Maintenance treatment with sintilimab (every three weeks) for up to one year was allowed based on the patient’s decision.

**Endpoints**

The primary endpoint was the proportion of patients achieving a major pathological response (MPR) at the time of surgery. Secondary endpoints included the pathological complete response (pCR) rate; objective response rate (ORR), defined as the proportion of patients with complete or partial response; two-year disease-free survival (DFS) rate, the proportion of patients without disease recurrence, metastasis, or death at two years; two-year overall survival (OS) rate, the proportion of patients alive at two years; and safety. Exploratory endpoints included novel immune biomarkers and the impact of sintilimab maintenance on the two-year DFS and OS.

**PD-L1 Immunohistochemistry**

Tumor tissue samples were obtained to assess PD-L1 expression before randomization. PD-L1 immunohistochemistry was performed with a PD-L1 immunohistochemistry E1L3N assay kit (Amoy Diagnostics, Xiamen, China). Tumor proportion score (TPS) was used to determine PD-L1 expression. TPS was defined as the percentage of tumor cells with partial or complete membrane staining.

**Assessments**

Patients underwent computed tomography scans at the baseline, within two weeks before surgery, one-month after surgery, and then every 3-6 months until two years after surgery. Comprehensive imaging examinations were performed at the baseline to exclude patients with distant metastases. The radiological response was assessed by the radiologists using RECIST. The radiologists were not aware of the grouping information.

The pathological stage was evaluated from the resected specimens according to the AJCC. The local pathologists without knowledge of the grouping information reviewed the resected specimens to assess the percentage of residual tumors. The samples were stained with hematoxylin-eosin using routine procedures. MPR was defined as 10% or less residual viable tumor in the surgically resected specimen. pCR was defined as no viable tumor in the resected specimen, including all the sampled regional lymph nodes.^5-7^

Adverse events (AEs) were monitored during the study period. The National Cancer Institute Common Terminology Criteria for Adverse Events (version 5.0) was used to assess the severity of each AE. During the first 30 days after the operation, postoperative complications and mortality were monitored and stratified using the Clavien-Dindo classification.^8^

**Statistical Analysis**

According to a three-stage group sequential randomized trial design, a sample size of N=102 (51 per arm) was required to detect an improvement in the MPR rate from 50% in the two-cycle arm to 70% in the three-cycle arm, considering a 10% attrition rate. The target power was 80%, with a two-sided type I error of 0.05. The PASS (version 11.0) was used to calculate the sample size.

Two interim analyses were planned when 33.3% and 66.7% of patients were recruited. The O’Brien–Fleming group sequential design was used with p-values of 0.000207, 0.01189, and 0.037903 in the two planned interim and final analyses, respectively. To account for futility, an unplanned interim analysis was performed after 60 patients were enrolled. The interim results were reviewed by the DSMB in December 2021. The DSMB recommended termination of the trial because the MPR rates were lower than predicted. It was unlikely that the three-cycle treatment led to a significant increase in MPR compared with the two-cycle treatment using the planned sample size.

The efficacy population included all randomized patients who underwent surgery. The safety population included all patients administrated one or more doses of the study treatment. The Mann-Whitney U test was used to compare continuous variables, and the χ^2^ test or Fisher’s exact test was used for categorical variables. The Clopper-Pearson method was used to estimate the associated 95% confidence intervals (CIs). Correlations between the pathological and radiological responses were analyzed using Spearman’s correlation. Exploratory subgroup analyses were conducted for the efficacy endpoints. A multivariable logistic regression model was used to analyze the association between clinical factors and MPR. The DFS and OS curves were estimated using Kaplan-Meier method, and a two–sided log–rank test were used to compare the difference between each treatment arm. The hazard ratio (HR) and associated 95% CIs were calculated. *P* <0 .05 indicated statistical significance. Statistical analyses were conducted with SPSS (version 24.0) and GraphPad Prism (version 9.0).

**References:**

1. Detterbeck, F.C., Boffa, D.J., Kim, A.W. & Tanoue, L.T. The eighth edition lung cancer stage classification. *Chest* **151**, 193–203 (2017).

2. Goldstraw, P. et al. The IASLC lung cancer staging project: proposals for revision of the TNM stage groupings in the forthcoming (eighth) edition of the TNM classification for lung cancer. *J. Thorac. Oncol.* **11**, 39-51 (2016).

3. Eisenhauer, E.A. et al. New response evaluation criteria in solid tumours: revised RECIST guideline (version 1.1). *Eur. J. Cancer* **45**, 228–247 (2009).

4. Oken, M.M. et al. Toxicity and response criteria of the Eastern Cooperative Oncology Group. *Am. J. Clin. Oncol.* **5**, 649–655 (1982).

5. Hellmann, M.D. et al. Pathological response after neoadjuvant chemotherapy in resectable non-small cell lung cancers: proposal for the use of major pathological response as a surrogate endpoint. *Lancet Oncol.* **15**, e42–e50 (2014).

6. Travis, W.D. et al. IASLC multidisciplinary recommendations for pathologic assessment of lung cancer resection specimens after neoadjuvant therapy. *J. Thorac. Oncol.* **15**, 709-740 (2020).

7. Pataer, A. et al. Histopathologic response criteria predict survival of patients with resected lung cancer after neoadjuvant chemotherapy. *J. Thorac. Oncol.* **7**, 825–832 (2012).

8. Dindo, D., Demartines, N. & Clavien, P.A. Classification of surgical complications: a new proposal with evaluation in a cohort of 6336 patients and results of a survey. *Ann. Surg.* **240**, 205-213 (2004).


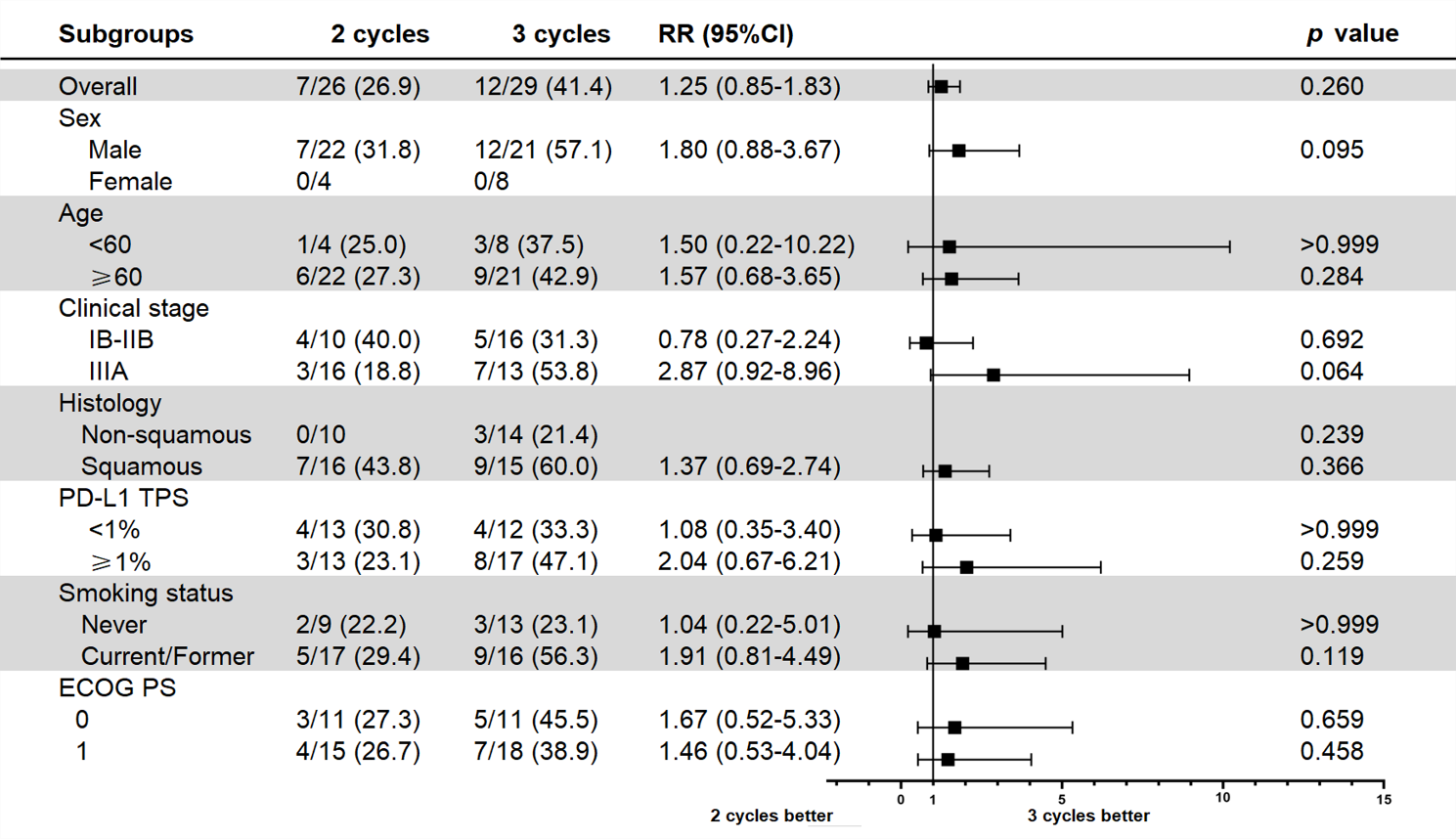


Figure. S1.

Forest plot for baseline characteristics. PD-L1, programmed cell death ligand-1; TPS, tumor proportion score; ECOG PS, Eastern Cooperative Oncology Group performance status; RR, risk ratio.


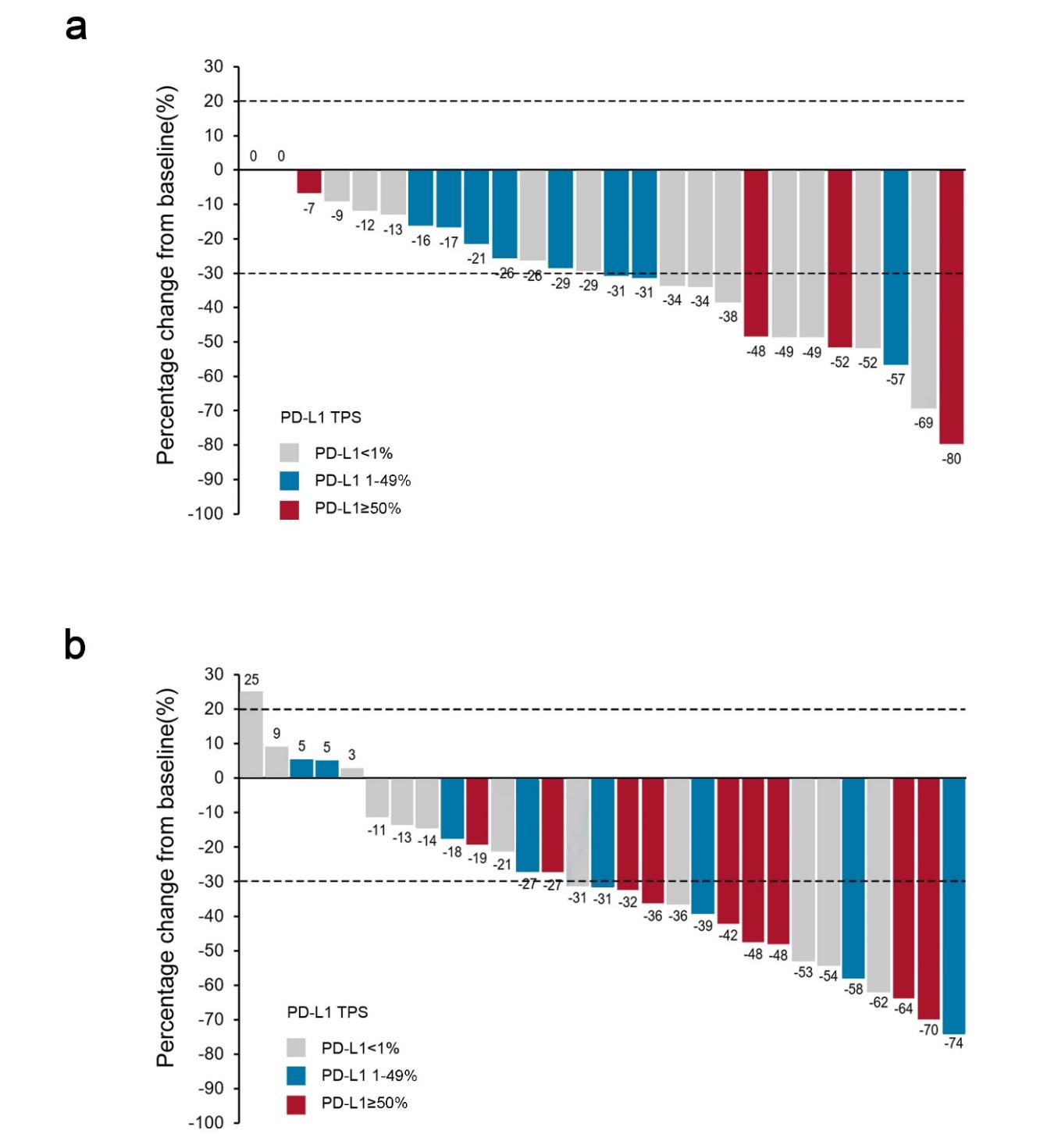


Figure. S2.

Waterfall plot. The percentage change in tumor size after (a) two cycles or (b) three cycles of neoadjuvant sintilimab plus chemotherapy (n=26) via RECIST v1.1. The dashed lines represented PD (20% increase in tumor size) and PR (30% reduction in tumor size). PD-L1, programmed cell death ligand-1; TPS, tumor proportion score.


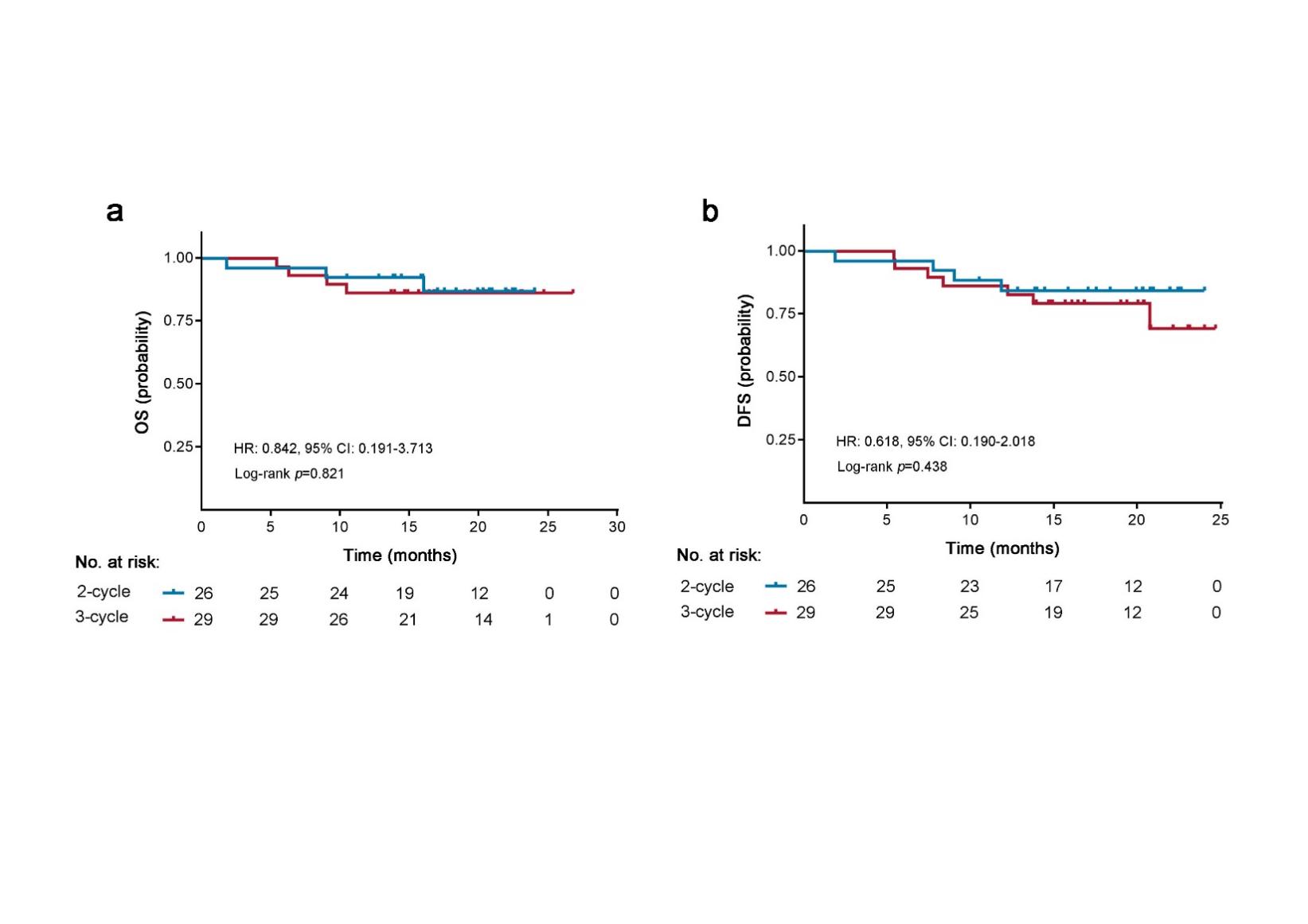


Figure. S3.

Kaplan-Meier curves for (a) OS and (b) DFS. HR, hazard ratio; OS, overall survival; DFS, disease-free survival.


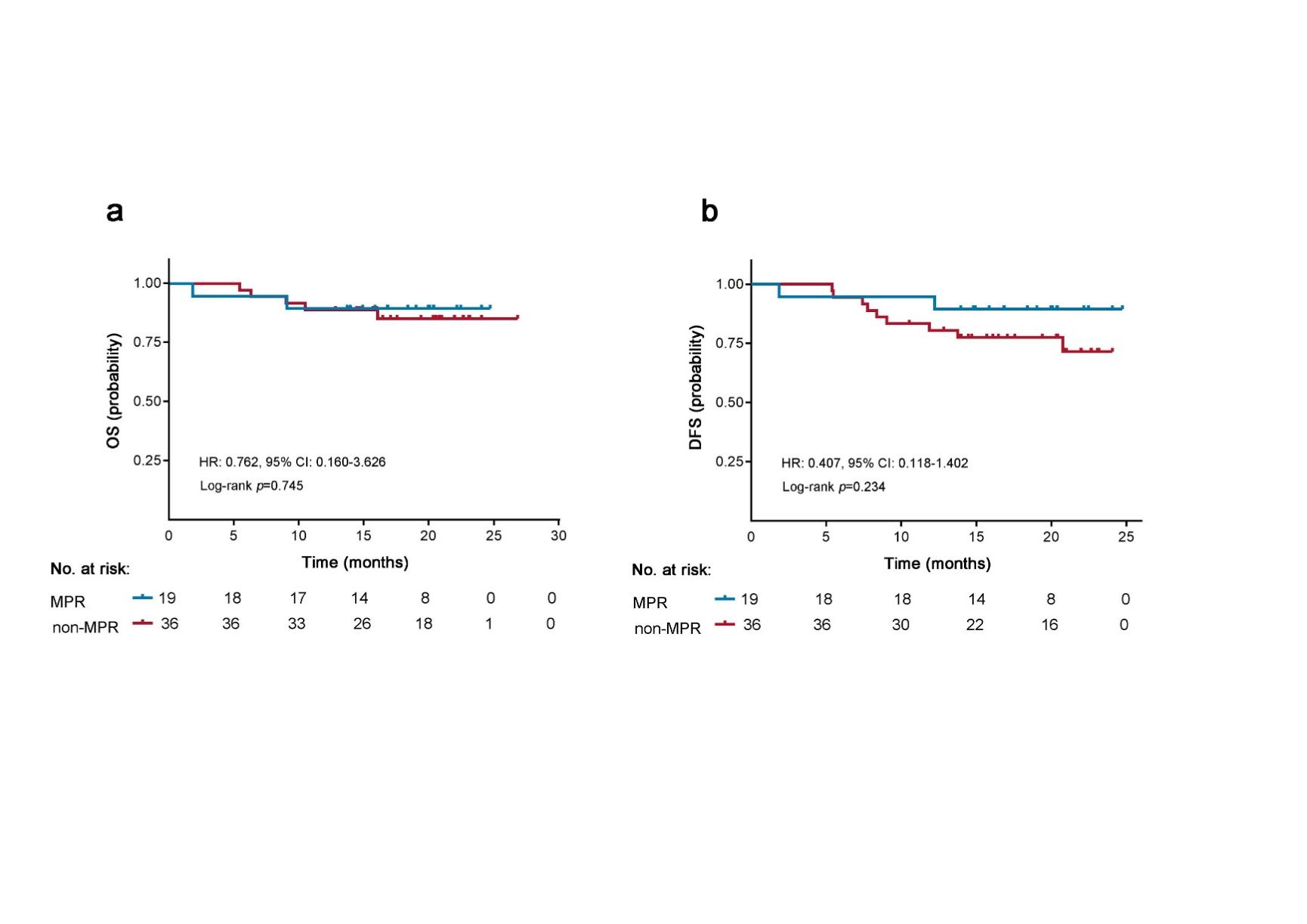


Figure. S4.

Kaplan-Meier curves for (a) OS and (b) DFS by pathological response. HR, hazard ratio; OS, overall survival; DFS, disease-free survival; MPR, major pathological response.

Table S1.

Baseline characteristics.

NOTE. Data are No. (%) or median (range).

Abbreviations: ECOG PS, Eastern Cooperative Oncology Group performance status; AJCC, American Joint Committee on Cancer; NOS, not otherwise specified; PD-L1, programmed cell death ligand-1; TPS, tumor proportion score.

Table S2.

Surgical outcomes and postoperative complications (n = 55).

NOTE. Data are No. (%) or median (range).

^1^ One patient in 2-cycle arm had grade V bronchial hemorrhage, which was considered unrelated to the study drug.

**Table S3.**

Baseline characteristics of two patients who underwent pneumonectomy in 3-cycle arm.

**Table S4.**

Surgical details of two patients who underwent pneumonectomy in 3-cycle arm.

^1^ The patient was found that the tumor invaded the right main pulmonary artery during the operation.

^2^ The patient was found that the tumor invaded the right main bronchus during the operation.

**Table S5.**

The major pathological response rate in 2-cycle and 3-cycle arms.

NOTE. Data are No. (%).

^1^ Three patients who did not comply with the protocol were excluded.

**Table S6.**

Detailed information of patients who underwent NGS testing.

**Table S7. Patient response.**

NOTE. Data are No. (%).

Abbreviations: MPR, major pathological response; pCR, pathological complete response; ORR, objective response rate.

**Table S8.**

Neoadjuvant treatment-related adverse events between the two- and three- cycle arms. ^1^

NOTE. Data are No. (%).

^1^ TRAEs of any grade occurred in ≥ 10% of patients in either group, or any TRAEs of grade≥ 3.

Abbreviations: TRAEs, treatment-related adverse events.

**Table S9.**

Adjuvant treatment-related adverse events between the two arms. ^1^

NOTE. Data are No. (%).

^1^ TRAEs of any grade occurred in ≥ 10% of patients in either group, or any TRAEs of grade≥ 3.

Abbreviations: TRAEs, treatment-related adverse events.

**Table S10.**

Details of five patients who had a progressive disease based on radiographic assessment.
