# Supplementary material for: Two vs three cycles of neoadjuvant sintilimab plus chemotherapy for resectable non-small-cell lung cancer: neoSCORE trial
Source: Signal Transduct Target Ther. 2023 Apr 10;8:146. doi: 10.1038/s41392-023-01355-1 (PMC10083171; doi:10.1038/s41392-023-01355-1)
Supplement: Supplementary file 2 — Protocol [file 41392_2023_1355_MOESM2_ESM.docx]

**Study protocol**

**Neoadjuvant of Sintilimab combined with Chemotherapy for Resectable NSCLC（neoSCORE）：A Prospective, Randomized, Open-Label, Single-Center Phase 2 Trial**

**Protocol number:** 2020-KYY-518052-0092

**Protocol version:** Version 6.0

**Sponsor:** The Second Affiliated Hospital, Zhejiang University School of Medicine, Hangzhou, Zhejiang, China

**Principal Investigator:** Junqiang Fan, Fuming Qiu

**TABLE OF CONTENTS**

**1　Study Background5**

1.1 Epidemiological Background5

1.2 Immune Checkpoint Inhibitors (ICIs)5

1.3 Study Drug (sintilimab)7

1.3.1 Mechanism of Action of Sintilimab7

1.3.2 Pharmacodynamics and Pharmacokinetics7

1.3.3 Sintilimab in NSCLC8

1.4 Risk/Benefit Assessment 10

**2 Objectives of the Study11**

2.1 Primary11

2.2 Secondary11

2.3 Exploratory11

**3 Study Design12**

3.1 Overall Design12

3.2 Study Design Schematic13

3.3 Study Population14

3.3.1 Inclusion Criteria14

3.3.2 Exclusion Criteria15

3.3.3 Discontinuation of Treatment17

3.3.4 Discontinuation from the Study17

3.4 Study Schedule18

3.4.1 Screening18

3.4.2 Visits during Therapy Period19

3.4.3 Safety Follow-up20

3.4.4 Survival Follow-up21

3.4.5 Subsequent Anti-tumor Treatment21

3.4.6 Treatment Discontinuation/Withdrawal from the Study22

3.4.7 Lost to Follow-up22

3.5 Study Treatment22

3.5.1 Study Drugs22

3.5.2 Treatments Administered23

**4 Statistical Considerations23**

4.1 Sample Size23

4.2 Statistical Analyses24

4.2.1 General Approach24

4.2.2 Randomization24

4.2.3 Efficacy Analyses24

4.2.3.1 Primary Efficacy Endpoint24

4.2.3.2 Secondary Efficacy Endpoint24

4.2.4 Safety Analyses25

4.2.4.1 Adverse Events25

4.2.4.2 Drug Exposure25

4.2.5 Baseline Characteristics of Subjects25

**5 Data Management and Confidentiality26**

5.1 Data Management26

5.1.1 Data Handling and Record Keeping26

5.1.2 Source Data/Document Access26

5.2 Protection of Subject Data26

**6 Subject Information and Informed Consent27**

**7 Adverse Events27**

7.1 Definition of Adverse Event27

7.2 Definition of Serious Adverse Event28

7.3 Criteria for Adverse Event Severity Judgment29

7.4 Recording Adverse Events32

7.4.1 Collection of Adverse Events and Time Period32

7.4.2 Follow-up of Adverse Events32

7.4.3 Adverse Event Records32

7.5 Expedited Reporting of SAEs and Pregnancies33

7.6 Treatment of Adverse Events35

7.6.1 Dose Adjustments for Adverse Events35

7.6.2 Treatment36

**8 References37**

**9 Appendices38**

Appendix 138

Appendix 239

Appendix 340

Appendix 453

**1　Study Background**

**1.1 Epidemiological Background**

Malignant tumor has become the biggest threat to human health and life. In 2018, 18.1 million new cancer cases were diagnosed worldwide and 9.6 million people died from cancer [1]. Lung cancer is the foremost cause of cancer-related deaths in China and across the globe. According to the cancer registry data from 2015, the morbidity and mortality of lung cancer in China were 572.6/million and 458.7/million [2].

Primary lung cancer is the most common malignancy worldwide. According to WHO classification, lung cancer can be classified into two main types, small cell lung cancer (SCLC) and non-small cell lung cancer (NSCLC). NSCLC accounts for about 75 to 80% of lung cancers. Surgery is the main approach to treat NSCLC. However, most of the patients have a locally advanced or metastatic disease at time of diagnosis and are not candidate for curative treatment. And the prognosis of patients who underwent radical surgery alone was poor due to postoperative recurrence and metastasis. The overall 5-year survival rate after surgery was only about 70% in patients with stage I NSCLC. Thus, to find effective perioperative therapies is an important direction in the study of NSCLC.

**1.2 Immune checkpoint inhibitors (ICIs)**

In recent years, immune checkpoint inhibitor therapy has become a research hot point gradually. In contrast to chemotherapy and targeted therapies, checkpoint inhibitors do not act directly on the tumor cell. ICIs block the inhibitory signaling and restore the antitumor activity of T cells, resulting in tumor cell recognition and killing [3]. Currently, immune checkpoint targets which have shown clear clinical efficacy include cytotoxic T lymphocyte antigen-4 (CTLA4) and programmed death-1/programmed death ligand-1 (PD-1/PD-L1) [4]. PD1/PD-L1 immune checkpoint inhibitors have good prospects for clinical application because of the relatively wide spectrum of indication and good tolerance [5].

Immunotherapy has produced impressive clinical results in the first line or second line treatment of advanced NSCLC. However, its role in the perioperative treatment requires further exploration. Some studies have shown that neoadjuvant immunotherapy can improve the clinical efficacy. At present, some published clinical trials, such as LCMC3 trial, NEOSTAR trial and NADIM trial, have reported the results of immunotherapy in neoadjuvant setting.

The LCMC3 trial aimed to assess the efficacy and safety of neoadjuvant atezolizumab in patients with stages IB-IIIA NSCLC. The primary endpoint was major pathological response (MPR; ≤10% viable tumor cells at surgery). Secondary endpoints included safety and correlation with PD-L1 expression, tumor mutational burden (TMB), and gene expression signatures. The MPR rate was 19%, and the pathologic complete response (pCR) rate was 5%. In terms of safety, 6 of 101 patients experienced grade 3/4 treatment-related adverse events. In addition, no significant associations between MPR and PD-L1 expression or TMB were observed.

The NEOSTAR trial compared the efficacy of neoadjuvant nivolumab and nivolumab + ipilimumab in patients with resectable NSCLC. Compared with nivolumab, nivolumab + ipilimumab resulted in higher pathologic complete response rates. In 44 randomized patients, MPR was observed in 22% of patients in the nivolumab arm and 38% of patients in the dual therapy arm. In 37 patients resected on trial, nivolumab and nivolumab + ipilimumab produced MPR rates of 24% (5/21) and 50% (8/16), respectively. Additionally, a previous study showed an MPR rate of 45% (9/20) after neoadjuvant nivolumab in patients with resectable NSCLC.

The NADIM trial was the first multi-center trial investigating the efficacy of neoadjuvant immuno-chemotherapy in stage IIIA patients. Eligible patients received three cycles of neoadjuvant nivolumab plus chemotherapy (paclitaxel + carboplatin). A total of 46 patients were enrolled. Of the 41 patients who had surgery, the MPR rate achieved 85.4% (35/41), and the pCR rate achieved 71.4% (25/41). The preliminary results are very encouraging and promising, but long-term results needed to be evaluated.

A prior study has evaluated sintilimab monotherapy as neoadjuvant therapy for resectable NSCLC [6]. The MPR rate achieved 40.5% (15/37), and the pCR rate achieved 16.2% (6/37). From the above, there have been some published studies about neoadjuvant immunotherapy in resectable NSCLC. However, the sample sizes of these existing studies were relatively small, and more high-grade evidence is needed.

**1.3 Study Drug (sintilimab)**

**1.3.1 Mechanism of Action of Sintilimab**

Sintilimab (R&D code: IBI308) is a recombinant fully human immunoglobulin G4 (IgG4) monoclonal antibody targeting PD-1 receptor. Sintilimab, a PD-1 inhibitor, blocks the PD-1/PD-L1 pathway through the specific binding to the PD-1 molecule on the surface of tumor infiltration lymphocytes. Multiple preclinical studies have shown the efficacy of sintilimab on blocking PD-1 pathway. The pharmacodynamics, pharmacokinetics, and toxicology were evaluated in preclinical researches. These studies showed that sintilimab has the characteristics of clear target and good stability. As well, sintilimab has shown promising activity in preclinical studies.

**1.3.2 Pharmacodynamics and Pharmacokinetics**

In September 2016, the phase Ia dose-escalation study was initiated (code: CIBI308A101-1a). This study enrolled patients with advanced solid tumors that failed standard treatment. Phase 1a study adopted the classical 3+3 dose escalation design, exploring safety and tolerance of 4 dose cohorts (1mg/kg, 3mg/kg, 200mg and 10mg/kg). After the completion of 1 mg/kg dose administration, subjects are randomized in a 1:1 ratio to either 3 mg/kg or 200 mg dose group for independent evaluations. Dose limiting toxicity (DLT) is observed for 28 days after the first dose for each dose group. After completion of DLT observation, subjects are treated with sintilimab Q2W (1 mg/kg, 3 mg/kg, or 10 mg/kg) or Q3W (200 mg) until progressive disease (PD), intolerable toxicity, withdrawal of informed consent, or other reasons requiring treatment discontinuation (whichever occurs first).

For the pharmacokinetic analysis, sintilimab exhibited linear pharmacokinetics over a 1–10 mg/kg dose range. The pharmacokinetic characteristics of sintilimab were similar to the marketed anti-PD-1 antibodies (e.g., nivolumab, pembrolizumab).

For the pharmacodynamic analysis, sintilimab (at a dose of 1 mg/kg) was able to rapidly (within 24 h) occupy PD-1 receptors on the surface of CD3-positive T cells in peripheral blood. Mean PD-1 receptor occupancy was≥ 95%, with high occupancy was maintained during the treatment. The mean PD-1 receptor occupancies were similar among the 4 dose cohorts, indicating that PD-1 receptor occupancy was not dose-dependent at the dose range of 1–10 mg/kg. Based on the previous pharmacokinetic/pharmacodynamic results and acceptable safety events, and taking into account potential individual differences, the recommended dose of sintilimab for further clinical study is 200 mg every 3 weeks.

**1.3.3 Sintilimab in NSCLC**

Currently, a number of phase II/III studies of the use sintilimab in the treatment of various solid tumors have been performed. The ORIENT-1 trial, a multicenter, single-arm, open-label, phase 2 study, assessed the activity and safety profile of sintilimab in Chinese patients with relapsed or refractory classical Hodgkin lymphoma [7]. According to the results of this trial, sintilimab was approved in China for the treatment of classical Hodgkin’s lymphoma in patients who have relapsed or are refractory after ≥ 2 lines of systemic chemotherapy.

As of October 16, 2018, a total of 540 patients from 5 studies have been treated with sintilimab, and the overall safety profile was similar to the marketed anti-PD-1 antibodies. Among 540 patients treated with sintilimab, the incidence of all-grade adverse effects was 86.1%. The adverse events that occurred in more than 10% of patients included fever, anemia, increased aspartate aminotransferase, increased alanine aminotransferase, fatigue, and decreased white blood cell count. The incidence of grade≥3 adverse effects was 30.6%. The grade ≥ 3 adverse events that occurred in more than 1% of patients included pulmonary infection, anemia, increased lipase, thrombocytopenia, pneumonia, neutropenia, hyponatremia, increased gamma-glutamyltransferase, infectious pneumonia, upper gastrointestinal hemorrhage and decreased lymphocyte count.

At present, a number of clinical studies have explored the use of sintilimab in the treatment of advanced NSCLC and showed a good prospect of application. The CIBI308A101 trial was reported at the 2018 CSCO annual meeting. Patients with advanced NSCLC who failed or became intolerant to first-line systemic standard treatment were enrolled in Cohort C (sintilimab 200 mg, every 3 weeks). The objective response rate (ORR) was 17.6% and the median progression-free survival (PFS) was 2.8 months. The results showed that sintilimab monotherapy had good antitumor activity in patients with advanced NSCLC. Treatment-naive patients with advanced non-squamous NSCLC were enrolled in Cohort D (sintilimab, pemetrexed, and cisplatin). The ORR was 68.4%, which confirmed the antitumor activity of sintilimab combined with chemotherapy in the first-line treatment of advanced non-squamous NSCLC. Treatment-naive patients with advanced squamous NSCLC were enrolled in Cohort E (sintilimab, gemcitabine, cisplatin). The ORR was 64.7%, and the disease control rate (DCR) was 100%, showing good efficacy and safety.

The ORIENT-11 trial was a phase III, randomized, double-blind, placebo-controlled study, aimed to assess the efficacy and safety of combining sintilimab with the pemetrexed/platinum regimen compared with pemetrexed/platinum alone in patients with advanced or recurrent non-squamous NSCLC. The median PFS was prolonged by 4 months. Combination therapy was associated with a 45% reduction in the risk of disease progression [8]. The ORIENT-12 trial included patients with advanced or recurrent squamous NSCLC. Patients received either sintilimab plus chemotherapy or chemotherapy alone. The median PFS was 5.5 months in the combo group and 4.9 months in the chemo group. Combination therapy was associated with a 46% reduction in the risk of disease progression [9].

A study of sintilimab combined with anlotinib in the first-line treatment of advanced NSCLC was reported at the 2019 WCLC meeting. The 6-month PFS rate was 93.8%, the ORR was 72.7%, and the DCR was 100%. sintilimab in combination with anlotinib has exhibited encouraging activity in patients with treatment-naive NSCLC, with good safety profile and tolerability [10].

In addition, some studies have confirmed that sintilimab can improve the survival benefit of resectable NSCLC patients in the neoadjuvant setting. A study of neoadjuvant sintilimab in resectable NSCLC was reported at the 2019 ASCO meeting. Treatment-naive patients with resectable NSCLC (stage IA–IIIB) received two cycles of sintilimab (200 mg, intravenously, day 1 out of 22). Operation was performed between day 29 and 43. A total of 40 patients enrolled, and the ORR rate was 20.0% (8/40). Among them, 37 underwent radical resection, 15 (40.5%) achieved MPR, including six (16.2%) with a pCR. The neoadjuvant sintilimab treatment achieved a high MPR rate, and was well tolerated [6].

**1.4 Risk/Benefit Assessment**

Based on the pharmacological mechanism of sintilimab and the clinical safety information from monoclonal antibodies with the same mechanism, it is predicted that the possible adverse events of sintilimab are mainly various immune inflammations caused by immune system activation, such as pneumonia, thyroiditis, hepatitis, dermatitis/skin lesions, etc. Based on the available clinical data of anti-PD-1 monoclonal antibodies, although the incidence of adverse reactions is high, the anti-PD-1 drugs were well tolerated. Only a small number of patients discontinued the medication due to adverse events. Also, most adverse events can be relieved after treatment. The investigators should pay special attention to the early clinical signs and symptoms of immune-related reactions due to the variable early symptoms of immune related adverse events. If an immune related adverse event occurred, corresponding treatments should be given and the dosage should be adjusted, so as to reduce the risk of drugs. At the same time, patients with autoimmune diseases should be excluded. During anti-PD-1 treatment, the activation of the immune system may lead to the aggravation of the preexisting disease.

The above data have demonstrated the efficacy and promising prospects of sintilimab in neoadjuvant treatment. The present study was designed to explore the efficacy and safety of neoadjuvant sintilimab plus platinum-doublet chemotherapy in resectable IB-IIIA NSCLC.

**2 Objectives of the Study**

NeoSCORE was a single-center, prospective, randomized, open-label, phase II study that aimed to evaluate different cycles of neoadjuvant sintilimab plus chemotherapy for resectable IB-IIIA NSCLC.

**2.1 Primary**

- To assess the MPR rate of different cycles of neoadjuvant sintilimab plus chemotherapy for resectable NSCLC

**2.2 secondary**

- To assess the pCR rate and ORR of different cycles of neoadjuvant sintilimab plus chemotherapy for resectable NSCLC
- To assess the 2-year disease-free survival (DFS) rate and 2-year overall survival (OS) rate of different cycles of neoadjuvant sintilimab plus chemotherapy for resectable NSCLC
- To assess the safety of different cycles of sintilimab and chemotherapy；

**2.3 Exploratory:**

- To explore predictive biomarkers for neoadjuvant immunotherapy plus chemotherapy；
- To assess the impact of sintilimab maintenance on the 2-year DFS and OS

**3 Study Design**

**3.1 Overall Design**

This trial is a single-center, prospective, randomized, open-label, phase II study that assesses the efficacy and safety of different cycles of neoadjuvant sintilimab combined with chemotherapy for resectable NSCLC. After the signing of the informed consent form (ICF), patients with treatment-naive resectable NSCLC who are confirmed eligible for the inclusion/exclusion criteria after screening will be randomly assigned to 2-cycle arm and 3-cycle arm (1:1).

**2-cycle arm:** Patients with non-squamous NSCLC (included adenocarcinoma, large cell carcinoma, and unspecified carcinoma) will receive two cycles of neoadjuvant sintilimab plus pemetrexed and carboplatin. Patients with squamous NSCLC will receive two cycles of neoadjuvant sintilimab plus nab-paclitaxel and carboplatin. Surgery will be performed within the fourth week (±7 days) after the last dose of neoadjuvant treatment. After operation, patients will receive two cycles of the same regimen. Radiotherapy will be administered based on clinical situations and pathological staging. Maintenance treatment with sintilimab (every three weeks) for up to one year is allowed according to the patient’s decision.

**3-cycle arm:** Patients with non-squamous NSCLC (included adenocarcinoma, large cell carcinoma, and unspecified carcinoma) will receive three cycles of neoadjuvant sintilimab plus pemetrexed and carboplatin. Patients with squamous NSCLC will receive three cycles of neoadjuvant sintilimab plus nab-paclitaxel and carboplatin. Surgery will be performed within the fourth week (±7 days) after the last dose of neoadjuvant treatment. After operation, patients will receive one cycle of the same regimen. Radiotherapy will be administered based on clinical situations and pathological staging. Maintenance treatment with sintilimab (every three weeks) for up to one year is allowed according to the patient’s decision.

Patients will undergo computed tomography scans at baseline, within two weeks before surgery, one month post-surgery, and then every 3-6 months until two years post-surgery. Comprehensive imaging examinations will be performed at the baseline to exclude patients with distant metastases. Baseline imaging will be performed within 28 days prior to the start of treatment. The primary endpoint is the MPR rate of different cycles of neoadjuvant sintilimab plus chemotherapy for resectable NSCLC. Secondary endpoints include the pCR rate, ORR, two-year DFS rate, two-year OS rate, and safety. Exploratory endpoints include novel immune biomarkers and the impact of sintilimab maintenance on the two-year DFS and OS.

Patients will be followed for safety (one month post-operation or three months after the first dose of sintilimab, whichever occurs later). And patients will be followed up for survival until withdrawal of consent, lost to follow-up, death or study closure.

This study will collect tumor tissue, blood, urine and stool samples to explore biomarkers in neoadjuvant immunotherapy. The samples will be analyzed by single-cell sequencing, multigene sequencing, flow cytometry, fecal microecological metagenomics and urine metabonomics.

The estimated duration of this study is 36 months.

**3.2 Study Design Schematic**


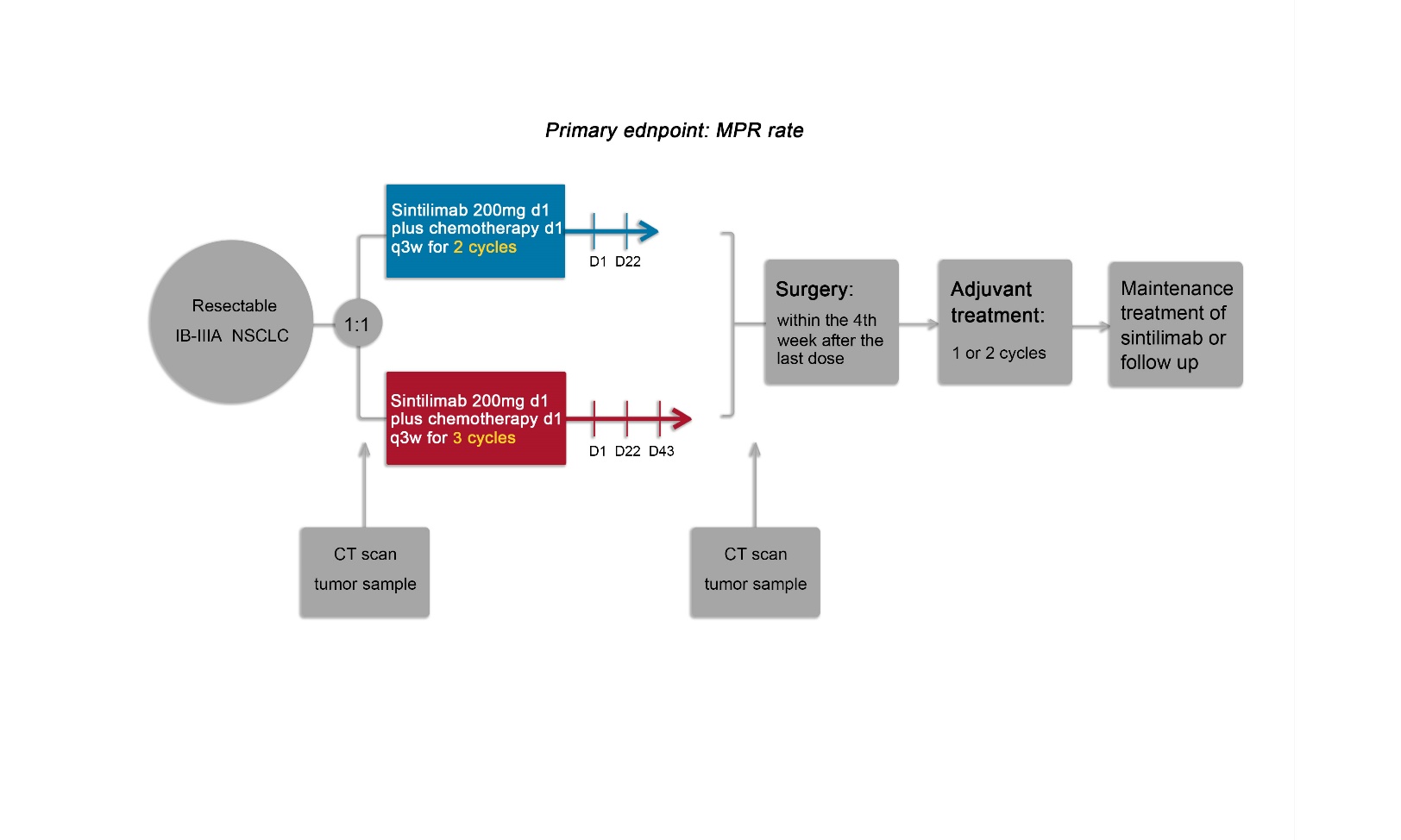


**3.3 　Study Population**

**3.3.1 Inclusion Criteria**

1. Patients signed written informed consent prior to any study-related procedures;

2. Aged 18-75 years old, male or female;

3. Cytologically or histologically proven NSCLC;

4. At least one measurable lesion (RECIST v1.1);

5. Treatment-naive patients with resectable ⅠB-ⅢA NSCLC (AJCC 8^th^ edition). The tumor resectability is assessed by thoracic surgeons;

6. ECOG performance status of 0 or 1;

7. Life expectancy > 6 months;

8. Adequate organ functions that meet the following criteria:

1) Absolute neutrophils count (ANC) ≥1.5 × 10^9^/L, platelets count (PLT) ≥100 ×10^9^/L, and hemoglobin (HB) >9g/dL without using granulocyte colony stimulating factor during the 14 days before the first dose of study treatment;

2) Total bilirubin (TBIL) ≤1.5 × ULN, ALT≤ 2.5 × ULN, AST≤ 2.5 × ULN, serum creatinine (sCr) ≤1.5 × ULN;

3) Coagulation function: INR≤1.5 or PT≤1.5 × ULN;

4) Thyroid function: thyroid-stimulating hormone (TSH) within the normal range;

9. Women of childbearing age must have negative urine or serum pregnancy tests within 3 days before the first dose (cycle 1 day 1). If the urine pregnancy test cannot be confirmed as negative, a serum pregnancy test is required.

**3.3.2 Exclusion Criteria**

1. Diagnosed with malignancies within 5 years prior to the first dose (excluding skin basal cell carcinoma, skin squamous cell carcinoma and/or carcinoma in situ with radical treatment);

2. Currently participating in an interventional clinical trial, or have received other investigational drugs or medical instruments within 4 weeks before the first dose;

3. Previous treatment with an anti-PD-1, anti-PD-L1, anti-PD-L2, or any other antibody or drug specifically targeting T-cell co-stimulation (e.g., CTLA-4, OX-40, CD137);

4. Patient had an active autoimmune disease which required systemic treatment (e.g., disease modifying drugs, corticosteroids, immunosuppressants) within 2 years before the first dose. Alternative therapies (e.g., thyroxine, insulin, physiological doses of corticosteroids for adrenal or pituitary insufficiency) were not considered as systemic treatment;

5. Undergoing systemic glucocorticoid treatment (excluding topical glucocorticoids for intranasal, inhalation or other routes) or any other immunosuppression therapies within 7 days before the first dose.

Note: physiological doses of glucocorticoids are permitted (prednisone≤10 mg/d or equivalent drugs)

6. Allogeneic organ transplantation (excluding corneal transplantation) or allogeneic hematopoietic stem cell transplantation;

7. Allergic to study drug components or excipients (sintilimab, pemetrexed, carboplatin, nab-paclitaxel);

8. Not fully recovered from toxicities and/or complications before the first dose;

9. History of human immunodeficiency virus (HIV) infection (HIV-1/2 antibody positive);

10. Untreated active Hepatitis B (HBsAg positive and HBV-DNA copies > ULN);

11. Active Hepatitis C (HCV-Ab positive and HCV-RNA level higher than the detection limit);

12. Received live vaccines within 30 days before the first dose;

Note: Injecting inactivated virus vaccines for seasonal influenza within 30 days before the first dose is allowed, nevertheless, intranasal attenuated vaccines are not permitted.

13. Pregnant or lactating women;

14. Serious or uncontrolled systemic diseases, such as:

1) Resting ECG has significant and uncontrolled abnormalities in rhythm, conduction or morphology, such as complete left bundle branch block, second degree of heart block and above, ventricular arrhythmia, and atrial fibrillation;

2) Unstable angina, congestive heart failure, chronic heart failure with New York Heart Association (NYHA) score ≥ grade Ⅱ;

3) Arterial thrombosis, embolism or ischemia within 6 months before recruitment, such as myocardial infarction, unstable angina, cerebrovascular accident, and transient ischemic attack;

4) History of noninfectious pneumonia requiring glucocorticoid treatment within 1 year before the first dose, or having active interstitial lung diseases currently;

5) Active pulmonary tuberculosis;

6) Active or uncontrolled infections requiring systemic treatment;

7) Liver diseases, such as cirrhosis, decompensated liver disease, and acute/chronic active hepatitis;

8) poorly controlled diabetes (fasting blood glucose (FBG)＞10mmol/L);

9) Urine routine test indicates that urinary protein ≥ ++, and 24 hours urinary protein ＞1.0 g;

10) Patients with mental disorders who are unable to cooperate with the treatment;

15.Medical histories, treatments or abnormal laboratory results that may interfere the study results or prevent patients from participating in the whole process of the study. Researchers consider that participating in the study is inappropriate for the patient or the patient has other potential risks so that unsuitable for the study.

**3.3.3 Discontinuation of Treatment**

Subjects can discontinue the study treatment at any time and for any reason. The investigators can decide whether to stop the study treatment when an adverse event occurs. In addition, investigators can discontinue any subject from the study treatment if the subject is not suitable for treatment, violates the study protocol or for other safety reasons.

The subject **MUST** discontinue the study treatment for any of the following reasons:

- The subject or their legal representatives request discontinuation of study treatment
- Occurrence of any adverse events requiring discontinuation of treatment specified in the protocol (Section 5.4)
- Occurrence of intercurrent illness preventing further treatment
- Investigator decision
- Subjects with a positive pregnancy test
- Subjects with poor compliance
- Investigator considers that the subject will be put at unnecessary risk if the study drug is continued based on the subject's condition
- Complete the prescribed treatment protocol

All subjects who discontinued the study drug should be followed up according to the protocol specified follow-up procedures, unless a subject has withdrawn informed consent to all study procedures.

**3.3.4 Discontinuation from the Study**

If the subject or their legal representatives withdraw the informed consent, the subject will be withdrawn from the study. If the subject withdraws from the study, he or she will no longer receive study treatment or planned visits. With the consent of the subjects, they can be followed up for survival after withdrawal from the study. If the subject is lost to follow-up, the subject will be withdrawn from the study.

**3.4 Study Schedule**

**3.4.1 Screening**

The following study procedures must be completed during the screening period (Day -28 to -1) to ensure the subject’s eligibility for the study:

- Sign ICF
- Inclusion/exclusion criteria
- Demographics, past medical history, previous and concomitant medications
- Vital signs, height and weight
- Physical examination
- ECOG PS score
- 12-lead ECG
- Hematology/blood biochemistry/urinalysis
- Coagulation profile
- Pregnancy test
- Thyroid function
- Myocardial enzyme spectrum
- HIV antibody, hepatitis B two and a half pairs (HBsAg, HBsAb, HBcAb, HBeAg, HBeAb), HCV antibody. If necessary, HBV-DNA or HCV-RNA testing can be performed.
- Tumor imaging evaluation
- Biopsy of fresh tumor tissue
- Biomarker blood, urine, and fecal samples collection

**3.4.2 Visits during Therapy Period**

**Neoadjuvant Period**

- Vital signs, height and weight
- ECOG PS score
- 12-lead ECG
- Hematology/blood biochemistry/urinalysis
- Thyroid function
- Myocardial enzyme spectrum
- If necessary, HBV-DNA or HCV-RNA testing can be performed
- Adverse event assessment
- Concomitant medications
- Administration of study drug
- Biomarker blood, urine, and fecal samples collection

**Surgery (within the fourth week [±7 days] after the last dose)**

- Vital signs, height and weight
- ECOG PS score
- 12-lead ECG
- Hematology/blood biochemistry/urinalysis
- Coagulation profile
- Thyroid function
- Myocardial enzyme spectrum
- HIV antibody, hepatitis B two and a half pairs (HBsAg, HBsAb, HBcAb, HBeAg, HBeAb), HCV antibody. If necessary, HBV-DNA or HCV-RNA testing can be performed.
- Adverse event assessment
- Concomitant medications
- Tumor imaging evaluation
- Surgical details (surgical approach, extent of resection, operation time, post-operative hospitalization, and post-operative complication are recorded.)
- Biopsy of fresh tumor tissue
- Biomarker blood, urine, and fecal samples collection

**Adjuvant Period**

- Vital signs, height and weight
- ECOG PS score
- 12-lead ECG
- Hematology/blood biochemistry/urinalysis
- Thyroid function
- Myocardial enzyme spectrum
- Adverse event assessment
- Concomitant medications
- Administration of study drug
- Biomarker blood, urine, and fecal samples collection

**3.4.3 Safety Follow-up**

A safety follow-up will be conducted on Day 30 post-operation or Day 90 after the first dose of sintilimab (whichever occurs later). The safety follow-up covers the following:

- Vital signs
- Physical examination
- ECOG PS score
- 12-lead ECG
- Hematology/blood biochemistry/urinalysis
- Coagulation profile
- Thyroid function
- Myocardial enzyme spectrum
- Adverse event assessment
- Subsequent anti-tumor treatment
- Biomarker blood, urine, and fecal samples collection

**3.4.4 Survival Follow-up**

After the safety follow-up phase, patients enter the survival follow-up phase. Patients are followed with voice or in-person contact every 3 to 6 months. The investigators should try their best to get as much information (data about survival and subsequent anti-tumor treatment) as possible from the patients. For patients who discontinued the study treatment for reasons other than disease progression, information about disease progression should be obtained whenever possible. Patients will be followed until death or the end of the study.

**3.4.5 Subsequent Anti-tumor Treatment**

Data on new anti-tumor therapies after the last study treatment will be collected whenever possible. Patients will enter the survival follow-up phase after the initiation of new anti-cancer therapies. See section 3.4.4 for more details about survival follow-up.

**3.4.6 Treatment Discontinuation/Withdrawal from the Study**

Subjects who discontinued treatment/withdrew from the study prior to completing the study protocol should be encouraged to be followed for protocol specified follow-up procedures.

When the subject discontinues treatment/withdraws from the study, all procedures applicable to the end of treatment should be performed. Any adverse events at the time of treatment discontinuation/withdrawal from the study should be followed up according to the safety requirements in section 7.4 (recording of adverse events). If the subject discontinues treatment/withdraws from the study for reasons other than disease progression, imaging evaluation should be performed at the end of treatment.

Treatment could be stopped for the subjects who have completed the treatment specified in the study protocol. After stopping treatment, the subjects should return to the research center for safety follow-up visit, and then enter the survival follow-up phase.

**3.4.7 Lost to Follow-Up**

If the subject fails to return to the clinic for the planned visit and/or could not be contacted by the research team, the research team must make every effort to contact the subject and reschedule the missed visit.

**3.5 Study Treatment**

**3.5.1 study drugs**

- Sintilimab: Innovent Biologics (Suzhou); specifications: 10 ml/branch.
- Pemetrexed: at the discretion of the study investigators.
- Nab-paclitaxel: at the discretion of the study investigators.
- Carboplatin: at the discretion of the study investigators.

**3.5.2 Treatments Administered**

**Sintilimab:** 200 mg IV infusion on day 1 of each 3-week cycle. In 2-cycle arm, patients will receive 2 cycles of sintilimab before surgery, followed by 2 cycles of sintilimab after surgery. In 3-cycle arm, patients will receive 3 cycles of sintilimab before surgery, followed by 1 cycle of sintilimab after surgery. Maintenance treatment with sintilimab (every three weeks) for up to one year is allowed according to the patient’s decision.

**Chemotherapy:**

- **Non-squamous NSCLC (included adenocarcinoma, large cell carcinoma, and unspecified carcinoma):** pemetrexed will be administered at a dose of 500 mg/m^2^ intravenously on day 1 of each 3-week cycle. Carboplatin will be administered at a dose of AUC 5 intravenously on day 1 of each 3-week cycle. In 2-cycle arm, patients will receive 2 cycles of chemotherapy before surgery, followed by 2 cycles of the same regimen after surgery. In 3-cycle arm, patients will receive 3 cycles of chemotherapy before surgery, followed by 1 cycle of the same regimen after surgery.
- **Squamous NSCLC:** nab-paclitaxel will be administered at a dose of 260 mg/m^2^ intravenously on day 1 of each 3-week cycle. Carboplatin will be administered at a dose of AUC 5 intravenously on day 1 of each 3-week cycle. In 2-cycle arm, patients will receive 2 cycles of chemotherapy before surgery, followed by 2 cycles of the same regimen after surgery. In 3-cycle arm, patients will receive 3 cycles of chemotherapy before surgery, followed by 1 cycle of the same regimen after surgery.

**4 Statistical Considerations**

**4.1 Sample Size**

According to a three-stage group sequential randomized trial design, a sample size of N=102 (51 per arm) will be required to detect an improvement in the MPR rate from 50% in the two-cycle arm to 70% in the three-cycle arm, considering a 10% attrition rate. The target power is 80%, with a two-sided type I error of .05. A stage group sequential method using O’Brien-Fleming boundaries will be followed, with p-values of 0.000207, 0.01189, and 0.037903 corresponding to 33%, 66%, and 100% of the patients enrolled, respectively.

**4.2 Statistical Analyses**

**4.2.1 General Approach**

Continuous variables will be expressed as mean, standard deviation, minimum, median and maximum, categorical variables as percentages and frequency distributions, unless otherwise stated.

**4.2.2 Randomization**

Patients who meet all the inclusion criteria and none of the exclusion criteria will be informed about the difference between two therapeutic regimens. After signing the informed consent form, the patient will be recruited in this study. This study is stratified-block randomized, with a block size of 6, using PD-L1 tumor proportion score as a stratification factor (<1% vs ≥1%). After enrolled, the patients are randomly allocated to the two groups accordingly, with their random numbers written on the study medical records.

**4.2.3 Efficacy Analyses**

**4.2.3.1 Primary Efficacy Endpoint**

- Major pathologic response (MPR) rate: the proportion of patients having ≤10% visible tumor cells in the surgical resected specimen.

$MPR=\frac{Number of subjects achieving MPR}{Total number of subjects}*100\%$, with its 95% CI calculated using binomial distribution.

**4.2.3.2 Secondary Efficacy Endpoint**

- Pathology complete response (pCR) rate: the proportion of patients having no visible tumor cells in the surgical resected specimen.
- Objective response rate (ORR): the proportion of patients having a complete response (CR) or a partial response (PR), measured by RECIST 1.1.
- Two-year disease-free survival (DFS) rate: the proportion of patients without disease recurrence, metastasis, or death at two years.
- Two-year overall survival (OS) rate: the proportion of patients alive at two years.

**4.2.4 Safety Analyses**

Safety analysis will include all patients who are randomized and received at least one dose of study treatment. Safety assessments will include adverse events (AEs), clinical laboratory tests, vital signs, and electrocardiograms.

**4.2.4.1 Adverse Events**

Verbatim description of adverse events will be mapped to MedDRA thesaurus terms and graded according to NCI CTCAE v5.0. All adverse events will be summarized by treatment arm and NCI CTCAE grade. The clinical laboratory tests, ECOG, vital signs, physical examinations, and electrocardiograms will be summarized by treatment arm and NCI CTCAE grade. The observed values and the relative changes from their baseline values will be recorded.

**4.2.4.2 Drug Exposure**

The exposure (included medication compliance) and duration (cycles) of the study drug in the subjects during the study period will be summarized. The dose adjustments during treatment and the number of dose adjustments will be recorded.

**4.2.5 Baseline Characteristics of Subjects**

The following baseline characteristics of the subjects will be descriptively summarized: demographic characteristics (gender, age); tumor diagnosis and treatment information (pathological diagnosis, clinical stage, previous treatment); baseline tumor detection (number of target lesions and non-target lesions, their location and total long diameter, etc.); other baseline information: height and weight (body mass index, body surface area), vital signs, ECOG PS score, laboratory findings, previous/concomitant/new combination medications, etc.

**5 Data Management and Confidentiality**

**5.1 Data Management**

**5.1.1 Data handling and record keeping**

Documents (protocol and protocol amendments, completed eCRF, signed ICF, etc.) in the clinical trial should be retained and managed in accordance with the requirements of GCP. The research center should keep these documents for 5 years after the end of the study. Safety and environmental risks should be considered when storing documents.

**5.1.2 Source Data/Document Access**

The investigator agrees that the relevant authorized regulatory authorities have direct access to all study-related documents, including the medical records of the subjects.

**5.2 Protection of Subject Data**

All records about the identity of the subjects are confidential, and these materials will not be made publicly available to the extent permitted by the applicable laws and regulations. Any public reports of this study will not disclose the identifying information of the subjects.

Take preventive measures to ensure the confidentiality of documents and prevent the identification of subjects. However, under special circumstances, some people may see the genetic data and personal identification code of a subject. For example, in the case of medical emergency, the sponsor, its representative doctors or investigator will know the subject's identification code and have access to the subject's genetic data. In addition, relevant authorities may require access to relevant documents.

**6 Subject Information and Informed Consent**

Before the initiation of any study procedure, the ICF is used to explain the possible risks and benefits of this study to potential participants. The language of ICF should be simple and easy to understand. The ICF statement should clarify that the ICF is signed voluntarily, and the risks and benefits that may be brought by participating in this study should be clear, and the subjects can withdraw from the study at any time. Only after the investigator has fully explained the details of the study, the subjects' questions have been satisfactorily answered, given enough time to consider, and obtained the written consent of the subjects or their legal representatives, can the subjects be included in the group. All signed ICFs must be kept in the investigator's file or in the subject folder.

The investigator is responsible for explaining the contents of the ICF to the subjects, and obtaining the ICF which was signed and dated by the subjects or their legal representatives before the start of the study. After signing, the investigator should send a copy of the signed ICF to the subjects. The investigators need to record the process of informed consent in the study source documents.

**7 Adverse Events**

**7.1 Definition of Adverse Event**

Adverse event (AE) is defined as any untoward and unexpected medical event that occurred since the signing the ICF, regardless of whether it has a causal relationship with the study drug. AEs include but are not limited to the following situations:

- - - - The aggravation of the original (before entering the clinical trial) medical condition/disease (including the aggravation of symptoms, signs and laboratory abnormalities);
      - Any new adverse medical condition (including symptoms, signs, and newly diagnosed diseases)
      - Clinically significant abnormal laboratory values or results.

**7.2 Definition of Serious Adverse Event**

The serious adverse event (SAE) refers to an adverse event that meets at least one of the following criteria:

- Results in death, excluding deaths due to disease progression from the studied indication.
- Is life-threatening ("Life threatening" in the definition means that the subject is at risk of death when this AE occurs, excluding the AE that may cause death if the event is aggravated).
- Requires inpatient hospitalization or prolongation of hospitalization, excluding the following:
- Rehabilitation institutions
- Nursing homes
- Routine emergency room admissions
- Same-day surgery (e.g., outpatient/same-day/ambulatory surgery)
- Hospitalization or prolonged hospitalization unrelated to the deterioration of AE is not an SAE. For example, hospitalization due to the original disease and there was no new adverse event or aggravation of the original disease (e.g., to examine laboratory abnormalities that persisted before the clinical trial); hospitalization for other reasons (e.g., routine annual physical examination); hospitalization specified in the protocol during the clinical trial (e.g., procedures according to the requirements of the protocol); elective hospitalization unrelated to the deterioration of adverse events (e.g., elective surgery); scheduled treatment or surgery should be recorded in the whole trial protocol and/or the baseline data of the subject; hospitalization only for using blood products due to the use of blood products.
- Results in persistent or significant disability/incapacity.
- Results in congenital abnormalities/birth defects.
- Other important medical events: defined as events that jeopardize the subject or require medical intervention to prevent the occurrence of any of the above situations.

**7.3 Criteria for AE Severity Judgment**

The investigators will assess all AEs according to the NCI CTCAE v5.0. All adverse events which change CTCAE grade will be recorded on the adverse event form of the CRF. Each adverse event must be evaluated whether it is a serious adverse event.

Detailed scoring rules are described in the table below.

| **CTCAE v5.0** | **Grade 1** | Mild; asymptomatic or mild symptoms; clinical or diagnostic observations only; intervention not indicated |
| --- | --- | --- |
|  | **Grade 2** | Moderate; minimal, local, or noninvasive intervention indicated; limiting age-appropriate daily activities. |
|  | **Grade 3** | Severe or medically significant event requiring medication but not immediately life-threatening; hospitalization or prolongation of hospitalization indicated; disabling; limiting self-care ADL. |
|  | **Grade 4** | Life-threatening consequences; urgent intervention indicated. |
|  | **Grade 5** | Death related to AE. |
| **AE duration** | Record the start and stop dates of the adverse event. If less than 1 day, indicate the appropriate length of time and units. | |
| **correlation between AEs and study drug** | Did the Sponsor’s product cause the adverse event? A medically qualified investigator will be required to provide assessment of causality of the relationship between the study drug and the AE. The investigator’s signed/dated initials on the source document or worksheet that supports the causality noted on the AE form, ensures that a medically qualified assessment of causality was done. This signed document must be retained for the required regulatory time frame. The criteria below are intended as reference guidelines to assist the investigator in assessing the likelihood of a relationship between the test drug and the adverse event based upon the available information.  The following components are to be used to assess the relationship between the study drug and the AE; the greater the correlation with the components and their respective elements (in number and/or intensity), the more likely the study drug caused the adverse event; | |
|  | **Exposure** | Is there evidence that the subject was actually exposed to study drug, such as: reliable history, acceptable compliance assessments (pill count, diary, etc.), expected pharmacological effect, or measurements of drug/metabolite in bodily specimen? |
|  | **Time course** | Did the AE follow in a reasonable temporal sequence from administration of the study drug?  Is the time of onset of the AE compatible with a drug-induced effect (applies to studies with investigational medicinal product)? |
|  | **Likely cause** | Is the AE not reasonably explained by another etiology such as underlying disease, other drugs/vaccines, or other host or environmental factors？ |
|  | **Dechallenge** | Was study drug discontinued or dose/exposure/frequency reduced?  If yes, did the AE resolve or improve?  If yes, this is a positive rechallenge. If no, this is a negative rechallenge.  Note: This criterion is not applicable if: (1) the AE resulted in death or permanent disability; (2) the AE resolved/improved despite continuation of study drug; (3) the study is a single-dose drug study; (4) the study drug is only used one time |
|  | **Rechallenge** | Was the subjet re-exposed to the study drug in this study？  If yes, did the AE recur or worsen?  If yes, this is a positive rechallenge. If no, this is a negative rechallenge.  Note: This criterion is not applicable if: (1) the initial AE resulted in death or permanent disability, or (2) the study is a single-dose study, or (3) the study drug is only used one time  Note: If a rechallenge is planned for an adverse event which was serious and which may have been caused by the sponsor’s product, or if re-exposure to the sponsor’s product poses additional potential significant risk to the subject, then the rechallenge must be approved in advance by the sponsor clinical director as per dose modification guidelines in the protocol. |
|  | **Consistency with study treatment profile** | Is the clinical/pathological presentation of the AE consistent with previous knowledge regarding the sponsor’s product or drug class pharmacology or toxicology? |
| The assessment of relationship will be reported on the case report forms/worksheets by an investigator who is a qualified physician according to his/her best clinical judgment, including consideration of the above elements. | | |
| **Document causality** | | **Use of the following scale of criteria as guidance (not all criteria must be present to be indicative of a study drug relationship).** |
| **Related** | | There is evidence of exposure to the sponsor’s product. The temporal sequence of the AE onset relate to the administration of the study drug is reasonable. The AE is more likely explained by the sponsor’s product than by another cause. |
| **Unrelated** | | Subject did not receive the study drug or temporal sequence of the AE onset relative to administration of the study drug is not reasonable or the AE is more likely explained by another cause than the study drug. (Also entered for a subject with overdose without an associated AE). |

**7.4 Recording Adverse Events**

The investigators should record AEs or SAEs using medical terminology/concepts. Spoken languages and abbreviations should be avoided. All AEs (including SAEs) should be recorded on the adverse event form in the electronic case report form (eCRF).

**7.4.1 Collection of Adverse Events and time period**

The investigators assess the information about AEs by asking the subjects non-inductive questions.

All AEs (including SAEs), whether observed by the investigator or spontaneously reported by the subjects, should be collected from the signing of the ICF through 30 days after the last dose.

Within 30 to 90 days after the last dose, the investigator should report all SAEs and AEs that are related to the study drug or procedures. After 90 days after the last dose, the investigator should report SAEs that are considered related to the study drug or procedures. If a new anti-tumor therapy is started within 90 days after the last dose, only SAEs related to the study drug need to be recorded subsequently.

**7.4.2 Follow-up of Adverse Events**

AEs should be followed up until they recover to baseline or grade 0 - 1 or the investigators consider that follow-up is not necessary for reasonable reasons (e.g., the AE cannot be recovered or has improved). If the AE cannot be recovered, the reasonable explanation should be recorded in the eCRF. Regardless of whether it is related to the study drug or not, the recovery of AEs or SAEs and their dates should be recorded in eCRF and medical records.

**7.4.3 Adverse Event Records**

The investigators should completely record any adverse event, including diagnosis (if there is no diagnosis, record symptoms and signs, including laboratory abnormalities), start and end date and time (if applicable), CTCAE severity grade and change (Grade 3 events and above events), whether it is a serious adverse event, the measures taken for the study drug, the treatment given due to AE and the outcome of the event, and the relationship between adverse events and study drug.

For serious adverse events, the investigators should also provide the date on which the AE meets the criteria for an SAE, the date on which the investigators became aware of the SAE, the basis on which the AE is determined as an SAE, the date of hospitalization, the date of discharge, the possible cause of death, the date of death, whether an autopsy was performed, the causality assessment with the study procedures, the causal assessment with other drugs, and other possible causes of SAE. The researcher should also provide the judgment basis of correlation and the description of SAE. In the SAE description, these information should also be included: the number, age, sex, height and weight of the subject; the indications and disease stages of the study drug and the related general conditions of the subjects; clinical course of SAE such as occurrence, development, prognosis and consequence; laboratory test results related to SAE (the examination time, unit and normal range must be provided); previous history, concomitant diseases, and their occurrence and duration of SAE; SAE related medication history, concomitant drugs and their treatment start date, duration, usage and dosage; the details information of the start time, duration, usage and dosage of the study drug.

**7.5 Expedited Reporting of SAEs and Pregnancies**

**Reporting of SAEs:**

The reporting period of SAE is from the signing of informed consent to 30 days (including 30 days) after the last dose. In case of an SAE during this period of time, whether it is an initial report or follow-up report, the investigator must immediately complete the Serious Adverse Event Report Form, and report the SAE to the Ethics Committee and the sponsor, and submit the completed form to China Food and Drug Administration (CFDA) and Innovent (drugsafety@innoventbio.com) no later than 24 hours after awareness. The specific processes are presented in Fig 1.

If serious adverse events occurred outside the above-mentioned period are considered related to study drug, they should also be reported.


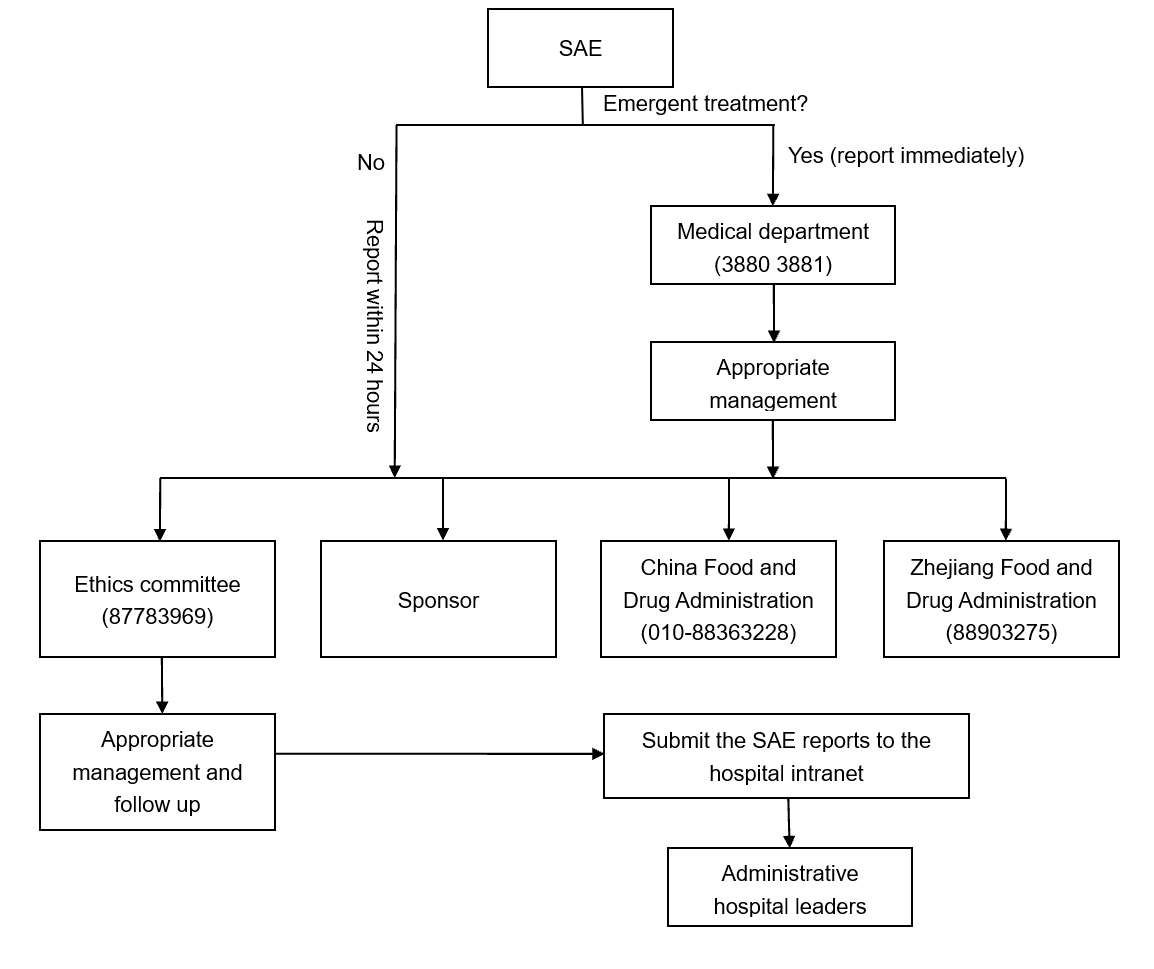


Fig 1. SAE reporting

**Pregnancy**

Similar drugs have been reported to have safety risks of embryotoxicity. All fertile subjects participating in the clinical trials must take effective contraceptive measures.

During the clinical trial, when the female subjects who were exposed to the study drugs became pregnant, the subjects should be excluded from the study, and the investigators should report to the sponsor and Innovent within 24 hours after awareness of the pregnancy, and complete the Innovent Clinical Trial Pregnancy Report/Follow-up Form. During the clinical trial, if the partner of the male subject exposed to the study drug becomes pregnant, the subject can continue participating in the clinical trial. The investigators should report to Innovent within 24 hours after awareness of the pregnancy, and complete the Innovent Clinical Trial Pregnancy Report/Follow-up Form.

The investigators should continuously monitor the subjects who are pregnant and follow up the pregnancy outcome until 8 weeks after the delivery, and report the outcome to the sponsor and Innovent.

If the result of pregnancy is stillbirth, spontaneous abortion, fetal malformation (any congenital abnormality/birth defect), or induced abortion for medical reasons, it should be regarded as SAE and should be reported according to the SAE procedures and time limits.

If the subject has a concurrent SAE during pregnancy, the SAE should be reported according to the SAE procedures.

**7.6 Treatment of adverse events**

**7.6.1 Dose adjustments for adverse events**

If an adverse event occurs during the study, the investigator should judge the possible relationship of reported AEs to the study drugs and adjust the drug dose. The subjects must have adequate hematologic, hepatic, and renal functions prior to dosing of study drug on Day 1, and all toxicities must have been resolved to NCI CTCAE grade 0-1 or baseline (excluding alopecia, fatigue). If the subject fails to meet the medication standard within the planned dosing interval due to adverse events, the next administration can be postponed. If it is necessary to suspend/permanently stop a certain study drug due to the toxicities, AEs or other reasons, other study drugs (sintilimab or chemotherapy) can be used alone when the corresponding medication standards are met.

All dosage modifications should be documented, including the reason and the methods used.

**7.6.2 Treatment**

The AEs related to the exposure of sintilimab may be of immunological etiology. This is because the mechanism of action of sintilimab is to block the interaction between PD-1 and PD-L1 and restore the activity of T cells, which may lead to autoimmune hyperfunction and immune related adverse reactions (irAEs). These irAEs may occur within a short time after the first administration or several months after the last administration of sintilimab, and may affect more than one body system at the same time, including immune-related pneumonia, diarrhea/enterocolitis, renal insufficiency, rash, hepatitis, endocrine disorders, and peripheral or central neuritis. Therefore, early detection and initiation of treatment are essential to reduce complications. Based on the existing clinical trial data, most irAEs are reversible and can be managed by interrupting the administration of sintilimab, glucocorticoid therapy and/or other supportive therapies. Once the subjects in this study have any of the above AEs, they should be monitored the symptoms and signs, meanwhile, the relevant examinations (such as bronchoscopy, endoscopy and skin biopsy) should be taken to identify the cause. If no alternative causes (e.g., disease progression, concomitant medication and infection) are identified and treatment with glucocorticoids and/or other immunosuppressive agents is required (except for endocrine events such as hyperthyroidism/hypothyroidism, hypophysitis, type 1 diabetes and adrenal insufficiency, which may not be treated with immunosuppressive therapy but are still considered to be related to immune hyperfunction caused by sintilimab), the above AEs should be considered to be related to hyperfunction of the immune system caused by sintilimab, and were diagnosed as irAEs. Based on the severity of the irAE, sintilimab treatment was suspended or permanently stopped, and glucocorticoids were given.

Before the initiation of any chemotherapy, colony-stimulating factors (such as GM-CSF) can be used to treat with the hematological toxicity, but preventive use before the first administration of chemotherapy is prohibited.

If the patient has surgery related adverse events, the investigator should treat them positively. All surgery related adverse events should be recorded.

**8 References**

[1] Bray F, Ferlay J, Soerjomataram I, et al: Global cancer statistics 2018: Globocan estimates of incidence and mortality worldwide for 36 cancers in 185 countries [J]. CA Cancer J Clin, 2018,68(6):394-424.

[2] Zheng R, Sun K, Zhang S, et al: Report of cancer epidemiology in China, 2015 [J]. Zhonghua Zhong Liu Za Zhi, 2019,41(1):19-28.

[3] McDermott DF, Atkins MB. Pd-1 as a potential target in cancer therapy [J]. Cancer Med, 2013,2(5):662-673.

[4] Barbee MS, Ogunniyi A, Horvat TZ, et al: Current status and future directions of the immune checkpoint inhibitors ipilimumab, pembrolizumab, and nivolumab in oncology [J]. Ann Pharmacother, 2015,49(8):907-937.

[5] NCCN Clinical Practice Guidelines in Oncology (NCCN Guidelines@). Non-Small Cell Lung Cancer. (version 5 2019- June 7 2019) [https://www.nccn.org/professionals/physician_gls/pdf/nscl.pdf]

[6] Gao S, Li N, Gao S, et al: Neoadjuvant PD-1 inhibitor (sintilimab) in NSCLC [J]. J Thorac Oncol, 2020,15(5):816-826

[7] Shi Y, Su H, Song Y, et al: Safety and activity of sintilimab in patients with relapsed or refractory classical Hodgkin lymphoma (ORIENT-1): a multicentre, single-arm, phase 2 trial [J]. Lancet Haematol, 2019,6(1):e12-e19.

[8] Yang Y, Zhou H, Zhang L. Response to Letter to the Editor: Efficacy and Safety of Sintilimab Plus Pemetrexed and Platinum as First-Line Treatment for Locally Advanced or Metastatic Nonsquamous NSCLC: A Randomized, Double-Blind, Phase 3 Study (ORIENT-11): [J]. J Thorac Oncol, 2020,15(12):e191-e192.

[9] Cai Z, et al: ORIENT-12: Sintilimab plus gemcitabine and platinum (GP) as first-line (1L) treatment for locally advanced or metastatic squamous non-small-cell lung cancer (sqNSCLC) [J]. Annals of Oncology, 2020, 31(4): S1142-S1215(suppl).

[10] Han B, Chu T, Zhong R, et al: Efficacy and Safety of Sintilimab with Anlotinib as First-Line Therapy for Advanced Non-Small Cell Lung Cancer (NSCLC) [J]. J Thorac Oncol, 2019, 14(10): S439(suppl).

**9 Appendices**

**Appendix 1: ECOG PS**

| Grade | Description |
| --- | --- |
| 0 | Asymptomatic, fully active, and able to carry on performance without restriction |
| 1 | Symptomatic, restricted in physically strenuous activity but ambulatory and able to carry out work of a light or sedentary nature, e.g., light house work, office work |
| 2 | Symptomatic, ambulatory and capable of all selfcare but unable to carry out any work activities, awake more than 50% of the time (< 50% of bed time during the day) |
| 3 | Symptomatic, capable of only limited selfcare, confined to bed or chair for > 50% of waking hours, but not yet bedridden |
| 4 | Completely disabled. Cannot carry on any selfcare. Totally confined to bed or chair |
| 5 | Dead |

**Appendix 2: Calculation of creatinine clearance and body surface area**

**1. Creatinine Clearance by Cockcroft-Gault Formula**

**1.1 Formula for calculating serum creatinine concentration (mg/dL):**

| Creatinine clearance in men (mL/min) = | | (140 - age) × (weight)^a^  72 × serum creatinine | |
| --- | --- | --- | --- |
| Creatinine clearance in women (mL/min) = | 0.85 × (140 - age) × (weight)^a^  72 × serum creatinine | |  |

**1.2 Formula for calculating serum creatinine concentration (μmol/L):**

| Creatinine clearance in men (mL/min) = | | (140 - age) × (weight)^a^  0.81 × serum creatinine | |
| --- | --- | --- | --- |
| Creatinine clearance in women (mL/min) = | 0.85 × (140 - age) × (weight)^a^  0.81 × serum creatinine | |  |

a: age in years and weight in kg.

**2. Stevenson formula for calculating body surface area**

Body surface area (m^2^) = 0.00616 height (cm) + 0.01286 weight (kg)− 0.1529

**Appendix 3: Response Evaluation Criteria in Solid Tumors (RECIST v1.1)**

**1. Measurability of Tumor at Baseline**

**1.1 Definitions**

At baseline, tumor lesions/lymph nodes will be categorized measurable or non-measurable as follows:

**1.1.1 Measurable**

Tumor lesions: Must be accurately measured in at least one dimension (longest diameter in the plane of measurement is to be recorded) with a minimum size of:

- 10 mm by CT scan (CT scan slice thickness no greater than 5 mm)
- 10 mm by conventional instruments in clinical exam (lesions which cannot be accurately measured by calipers should be recorded as non-measurable)
- 20 mm by chest X-ray
- Malignant lymph nodule: pathologically enlarged and measurable, single lymph nodule must be≥15mm in short axis by CT scan (CT scan slice thickness no greater than 5 mm). At baseline and during follow-up, only the short axis will be measured and followed.

**1.1.2 Non-measurable**

All other lesions, including small lesions (longest diameter <10 mm or pathological lymph nodule with ≥ 10 mm to < 15 mm short axis) as well as truly non-measurable lesions. Lesions considered truly non-measurable include: leptomeningeal disease, ascites, pleural or pericardial effusion, inflammatory breast disease, lymphangitic involvement of skin or lung, abdominal masses that cannot be diagnosed and followed by reproducible imaging techniques, and cystic lesions.

**1.1.3 Special considerations regarding lesion measurability**

Bone lesions, cystic lesions, and lesions previously treated with local therapy require particular comment:

**Bone lesions:**

1) Bone scan, PET scan or plain films are not considered adequate to measure bone lesions. However, these techniques can be used to confirm the presence or disappearance of bone lesions;

2) Lytic bone lesions or mixed lytic-blastic lesions, with identifiable soft tissue components, that can be evaluated by cross sectional imaging techniques such as CT or MRI can be considered as measurable lesions if the soft tissue component meets the definition of measurability described above.

3) Blastic bone lesions are non-measurable.

**Cystic lesions:**

1) Lesions that meet the criteria for radiographically defined simple cysts should not be considered as malignant lesions (neither measurable nor non-measurable) since they are, by definition, simple cysts;

2) Cystic lesions thought to represent cystic metastases can be considered as measurable lesions, if

they meet the definition of measurability described above. However, if noncystic lesions are present in the same subject, these are preferred for selection as target lesions.

**Lesions with prior local treatment:**

Tumor lesions situated in a previously irradiated area, or in an area subjected to other loco-regional therapy, are usually not considered measurable unless there has been demonstrated progression in the lesion. Study protocols should detail the conditions under which such lesions would be considered measurable.

**1.2 Specifications by methods of measurements**

**1.2.1. Measurements of lesions**

All measurements should be recorded in metric notation when clinically assessed. All baseline measurements of tumor lesions should be performed as close as possible to the treatment start and must be within 28 days (4 weeks) before the beginning of the treatment.

**1.2.2 Method of assessment**

The same method of assessment and the same technique should be used to characterize each identified and reported lesion at baseline and during follow-up. Imaging based evaluation should always be done rather than clinical examination unless the lesion being followed cannot be imaged but is assessable by clinical exam.

Clinical lesions: Clinical lesions will only be considered measurable when they are superficial and ≥10 mm diameter (e.g., skin nodules). For the case of skin lesions, documentation by color photography including a ruler to estimate the size of the lesion is suggested. When lesions can be evaluated by both clinical exam and imaging, imaging evaluation should be undertaken since it is more objective and may also be reviewed at the end of the study.

**Chest X-ray:** Chest CT is preferred over chest X-ray, particularly when progression is an important endpoint, since CT is more sensitive than X-ray, particularly in identifying new lesions. Lesions on chest X-ray may be considered measurable if they are clearly defined and surrounded by aerated lung.

**CT, MRI:** CT is the best currently available and reproducible method to measure lesions selected for response assessment. This guideline has defined measurability of lesions on CT scan based on the assumption that CT slice thickness is ≤5 mm. When CT scans have slice thickness greater than 5 mm, the minimum size for a measurable lesion should be twice the slice thickness. MRI is also acceptable in certain situations (e.g., for body scans).

**Ultrasound:** Ultrasound should not be used as a method of measurement to assess lesion size. Ultrasound examinations cannot be reproduced for review at a later date and, because they are operator dependent, it cannot be guaranteed that the same technique and measurements will be taken from one assessment to the next. If new lesions are identified by ultrasound in the course of the study, confirmation by CT or MRI is advised. If there is concern about radiation exposure at CT, MRI may be used instead of CT in selected instances.

**Endoscopy, laparoscopy:** The utilization of these techniques for objective tumor evaluation is not advised. However, they can be useful to confirm CR when biopsies are obtained or to determine relapse in trials where recurrence following CR or surgical resection is an endpoint.

**2. Tumor Response Evaluation**

**2.1 Evaluation of target lesions**

**Complete Response (CR):** Disappearance of all target lesions. Any pathological lymph nodes (whether target or non-target) must have reduction in short axis to < 10 mm.

**Partial Response (PR):** At least a 30% decrease in the sum of diameters of target lesions, taking as reference the baseline sum diameters.

**Progressive Disease (PD):** At least a 20% increase in the sum of diameters of target lesions, taking as reference the smallest sum on study (this includes the baseline sum if that is the smallest on study). In addition, the sum must also demonstrate an absolute increase of at least 5 mm. (Note: the appearance of one or more new lesions is also considered progression).

**Stable Disease (SD):** Neither sufficient shrinkage to qualify for PR nor sufficient increase to qualify for PD, taking as reference the smallest sum diameters while on study.

**2.2 Special notes on the assessment of target lesions**

Lymph nodes: Lymph nodes identified as target lesions should always have the actual short axis measurement recorded (measured in the same anatomical plane as the baseline examination), even if the nodes regress to below 10 mm on study. This means that when lymph nodes are included as target lesions, the "sum" of lesions may not be zero even if complete response criteria are met, since a normal lymph node is defined as having a short axis of < 10 mm Electronic CRFs or other data collection methods may therefore be designed to have target nodal lesions recorded in a separate section where, in order to qualify for CR, each node must achieve a short axis < 10 mm. For PR, SD and PD, the actual short axis measurement of the nodes is to be included in the sum of target lesions.

**Target lesions that become too small to measure:** While on study, all lesions (nodal and non-nodal) recorded at baseline should have their actual measurements recorded at each subsequent evaluation, even when very small (e.g., 2 mm). However, sometimes lesions or lymph nodes which are recorded as target lesions at baseline become so faint on CT scan that the radiologist may not feel comfortable assigning an exact measure and may report them as being "too small to measure". When this occurs, it is important that a value be recorded on the case report form. If it is the opinion of the radiologist that the lesion has likely disappeared, the measurement should be recorded as 0 mm. If the lesion is believed to be present and is faintly seen but too small to measure, a default value of 5 mm should be assigned. (Note: It is less likely that this rule will be used for lymph nodes since they usually have a definable size when normal and are frequently surrounded by fat such as in the retroperitoneum; however, if a lymph node is believed to be present and is faintly seen but too small to measure, a default value of 5 mm should be assigned in this circumstance as well). This default value is derived from the 5 mm CT slice thickness (but should not be changed with varying CT slice thickness). The measurement of these lesions is potentially non-reproducible, therefore providing this default value will prevent false responses or progressions based upon measurement error. To reiterate, however, if the radiologist is able to provide an actual measure, that should be recorded, even if it is below 5 mm.

**Lesions that split or coalesce on treatment:** When non-nodal lesions "fragment", the longest diameters of the fragmented portions should be added together to calculate the target lesion sum. Similarly, as lesions coalesce, a plane between them may be maintained that would aid in obtaining maximal diameter measurements of each individual lesion. If the lesions have truly coalesced such that they are no longer separable, the vector of the longest diameter should be the maximal longest diameter for the coalesced lesion.

**2.3 Evaluation of non-target lesions**

This section provides the definitions of the criteria used to determine the tumor response of non-target lesions. While some non-target lesions may actually be measurable, they need not be measured and instead should be assessed only qualitatively at the time points specified in the protocol.

**Complete Response (CR):** Disappearance of all non-target lesions and normalization of tumor marker level. All lymph nodes must be non-pathological in size (< 10 mm short axis).

**Non-Complete Response/Non-Progressive Disease (Non-CR/Non-PD):** Persistence of one or more non-target lesion(s) and/or maintenance of tumor marker level above the normal limits.

**Progressive Disease (PD):** Unequivocal progression of existing non-target lesions. Note: the appearance of one or more new lesions is also considered progression.

**2.4 Special notes on assessment of progression of non-target disease**

The concept of progression of non-target disease requires additional explanation as follows: When the subject also has measurable disease. In this setting, to achieve "unequivocal progression" on the basis of the non-target disease, there must be an overall level of substantial worsening in non-target disease such that, even in presence of SD or PR in target disease, the overall tumor burden has increased sufficiently to merit discontinuation of therapy. A modest "increase" in the size of one or more non-target lesions is usually not sufficient to qualify for unequivocal progression status. The designation of overall progression solely on the basis of change in non-target disease in the face of SD or PR of target disease will therefore be extremely rare.

When the subject only has non-measurable disease: This circumstance arises in some phase III trials when it is not a criterion of study entry to have measurable disease. The same general concepts apply here as noted above, however, in this instance there is no measurable disease assessment. Because worsening in non-target disease cannot be easily quantified (by definition: if all lesions are truly non-measurable) a useful test that can be applied when assessing subjects for unequivocal progression is to consider if the increase in overall disease burden based on the change in non-measurable disease is comparable in magnitude to the increase that would be required to declare PD for measurable disease: i.e. an increase in tumor burden representing an additional 73% increase in "volume" (which is equivalent to a 20% increase diameter in a measurable lesion). Examples include an increase in a pleural effusion from "trace" to "large", an increase in lymphangitic disease from "localized" to "widespread", or may be described in protocols as "sufficient to require a change in therapy". Examples include an increase in a pleural effusion from "trace" to "large", an increase in lymphangitic disease from "localized" to "widespread", or may be described in protocols as "sufficient to require a change in therapy". If "unequivocal progression" is seen, the subject should be considered to have had overall PD at that point. While it would be ideal to have objective criteria to apply to non-measurable disease, the increase must be substantial.

**2.5 New lesions**

The appearance of new malignant lesions denotes disease progression; therefore, some comments on detection of new lesions are important. There are no specific criteria for the identification of new radiographic lesions; however, the finding of a new lesion should be unequivocal. For example, progression should not be attributable to differences in scanning technique, change in imaging modality or findings thought to represent something other than tumor (for example, some "new" bone lesions may be simply healing or flare of pre-existing lesions). This is particularly important when the subject’s baseline lesions show partial or complete response. For example, necrosis of a liver lesion may be reported on a CT scan report as a "new" cystic lesion, which it is not.

A lesion identified on a follow-up study in an anatomical location that was not scanned at baseline is considered a new lesion and will indicate PD. An example of this is the subject who has visceral disease at baseline and while on study has a CT or MRI brain ordered which reveals metastases. The subject’s brain metastases are considered to be evidence of PD even if he/she did not have brain imaging at baseline.

If a new lesion is equivocal, for example because of its small size, continued therapy and follow-up evaluation will clarify if it represents truly new disease. If repeat scans confirm there is definitely a new lesion, then progression should be declared using the date of the initial scan.

While FDG-PET response assessments need additional study, it is sometimes reasonable to incorporate the use of FDG-PET scanning to complement CT scanning in assessment of progression (particularly possible "new" disease). New lesions on the basis of FDGPET imaging can be identified according to the following algorithm:

Negative FDG-PET at baseline, with a positive FDG-PET at follow-up is a sign of PD

based on a new lesion.

**No FDG-PET at baseline and a positive FDG-PET at follow-up:**

If the positive FDG-PET at follow-up corresponds to a new site of disease confirmed by CT, this is PD.

If the positive FDG-PET at follow-up is not confirmed as a new site of disease on CT, additional follow-up CT scans are needed to determine if there is truly progression occurring at that site (if so, the date of PD will be the date of the initial abnormal FDGPET scan).

If the positive FDG-PET at follow-up corresponds to a pre-existing site of disease on CT that is not progressing on the basis of the anatomic images, this is not PD.

**2.6 Missing assessments and in-evaluable designation**

When no imaging/measurement is done at all at a particular time point, the subject is not evaluable (NE) at that time point. If only a subset of lesion measurements are made at an assessment, usually the case is also considered NE at that time point, unless a convincing argument can be made that the contribution of the individual missing lesion(s) would not change the assigned time point response.

**2.7 Special notes on response assessment**

When nodal disease is included in the sum of target lesions and the nodes decrease to "normal" size (< 10 mm), they may still have a measurement reported on scans. This measurement should be recorded even though the nodes are normal in order not to overstate progression should it be based on increase in size of the nodes. As noted earlier, this means that subjects with CR may not have a total sum of "zero" on the eCRF.

In trials where confirmation of response is required, repeated "NE" time point assessments may complicate best response determination. The analysis plan for the trial must address how missing data/assessments will be addressed in determination of response and progression. For example, in most trials it is reasonable to consider a subject with time point responses of PR-NE-PR as a confirmed response.

Subjects with a global deterioration of health status requiring discontinuation of treatment without objective evidence of PD at that time should be reported as "symptomatic deterioration". Every effort should be made to document objective progression even after discontinuation of treatment. Symptomatic deterioration is not a descriptor of an objective response: it is a reason for stopping study therapy. The objective response status of such subjects is to be determined by evaluation of target and non-target disease as shown in Tables 1–3.

Conditions that define "early progression, early death and inevaluability" are study specific and should be clearly described in each protocol (depending on treatment duration, treatment periodicity).

In some circumstances it may be difficult to distinguish residual disease from normal tissue. When the evaluation of complete response depends upon this determination, a biopsy of the residual lesion is recommended before assigning a status of complete response. FDG-PET may be used to upgrade a response to a CR in a manner similar to a biopsy in cases where a residual radiographic abnormality is thought to represent fibrosis or scarring. The use of FDG-PET in this circumstance should be prospectively described in the protocol and supported by disease specific medical literature for the indication. However, it must be acknowledged that FDG-PET and biopsy may lead to false positive CR due to limitations of both approaches (resolution/sensitivity).

**Table 1. Time point response: subjects with target (with or without non-target) disease**

| Target Lesions | Non-Target Lesions | New Lesions | New Lesions |
| --- | --- | --- | --- |
| CR | CR | No | CR |
| CR | Non-CR/Non-PD | No | PR |
| CR | Not evaluated | No | PR |
| PR | Non-PD or not all evaluated | No | PR |
| SD | Non-PD or not all evaluated | No | SD |
| Not all evaluated | Non-PD | No | NE |
| PD  Any  Any | Any  PD  Any | Yes or No  Yes or No  Yes | PD  PD  PD |

Note: CR=complete response，PR=partial response，SD=stable disease，PD=progressive disease，NE=inevaluable

**Table 2. Time point response: subjects with non-target disease only**

| Non-Target Lesions | New Lesions | Overall Response |
| --- | --- | --- |
| CR | No | CR |
| Non-CR/Non-PD | No | Non-CR/Non-PD |
| Not all evaluated | No | Not evaluated |
| Unequivocal PD | Yes or No | PD |
| Any | Yes | PD |

Note: "Non-CR/non-PD" is preferred over "stable disease" for non-target disease. Since SD is increasingly used as endpoint for assessment of efficacy in some trials so to assign Non-CR/non-PD when no lesions can be measured is not advised.

For equivocal findings of progression (e.g., very small and uncertain new lesions; cystic changes or necrosis in existing lesions), treatment may continue until the next scheduled assessment. If at the next scheduled assessment, progression is confirmed, the date of progression should be the earlier date when progression was suspected.

**Table 3. Best overall response when confirmation of CR and PR required**

| Overall response first time point | Overall response subsequent time point | Best overall response |
| --- | --- | --- |
| CR | CR | CR |
| CR | PR | SD，PD 或 PR^a^ |
| CR | SD | SD provided minimum criteria for SD duration met, otherwise, PD |
| CR | PD | SD provided minimum criteria for SD duration met, otherwise, PD |
| CR | NE | SD provided minimum criteria for SD duration met, otherwise, NE |
| PR | CR | PR |
| PR | PR | PR |
| PR | SD | SD |
| PR | PD | SD provided minimum criteria for SD duration met, otherwise, PD |
| PR | NE | SD provided minimum criteria for SD duration met, otherwise, NE |
| NE | NE | NE |

Note: CR = complete response, PR = partial response, SD = stable disease, PD = progressive disease, and NE =Non-Evaluable. a: If a CR is truly met at first time point, then any disease seen at a subsequent time point, even disease meeting PR criteria relative to baseline, makes the disease PD at that point (since disease must have reappeared after CR). Best response would depend on whether minimum duration for SD is met. However, sometimes "CR" may be claimed when subsequent scans suggest small lesions were likely still present and in fact the subject had PR, not CR at the first time point. Under these circumstances, the original CR should be changed to PR and the best response is PR.

**2.8 Confirmatory Measurement/Duration of Response**

**2.8.1 Confirmation**

In non-randomized trials where response is the primary endpoint, confirmation of PR and CR is required to ensure responses identified are not the result of measurement error. In studies where stable disease or progression are the primary endpoints, confirmation of response is not required since it will not add value to the interpretation of trial results. In the case of SD, measurements must have met the SD criteria at least once after study entry at a minimum interval (in general not less than 6–8 weeks) that is defined in the study protocol.

**2.8.2 Duration of overall response**

The duration of overall response is measured from the time measurement criteria are first met for CR/PR (whichever is first recorded) until the first date that recurrent or PD is objectively documented (taking as reference for PD the smallest measurements recorded on study). The duration of overall complete response is measured from the time measurement criteria are first met for CR until the first date that recurrent disease is objectively documented.

**2.8.3 Duration of SD**

Stable disease is measured from the start of the treatment (in randomized trials, from date of randomization) until the criteria for progression are met, taking as reference the smallest sum on study (if the baseline sum is the smallest, this is the reference for calculation of PD). The clinical relevance of the duration of SD varies in different studies and diseases. If the proportion of subjects achieving stable disease for a minimum period of time is an endpoint of importance in a particular trial, the protocol should specify the minimal time interval required between two measurements for determination of stable disease.

**Note:** The duration of response and stable disease as well as the progression-free survival are influenced by the frequency of follow-up after baseline evaluation. It is not in the scope of this guideline to define a standard follow-up frequency. The frequency should take into account many parameters including disease types and stages, treatment periodicity and standard practice. However, these limitations of the precision of the measured endpoint should be taken into account if comparisons between trials are to be made

**Appendix 4: Principles of Pathologic Assessment of Primary Tumors**

**Lung Tumor Bed**

How to recognize the tumor bed, which is the area where the original pretreatment tumor was considered to be located

1. To identify the tumor bed, look for the presence of pleural retraction and palpate the intact specimen.

2. In cases where identification and, or orientation of the tumor are difficult, review of the pretherapy and preoperative computed tomography can be helpful.

3. Look for any identifying marks or stiches placed by the surgeon.

4. After the tumor bed has been identified, lung specimens should be sectioned in the plane that demonstrates the maximum dimension and best reveals the tumor bed and its relationship to the surrounding structures relevant for staging and the surgical resection margin(s).

5. Photograph the cut surface demonstrating the tumor bed and the adjacent structures. Save the images in the pathologic electronic records.

6. The gross size of the tumor bed should be assessed using a ruler to measure three-dimensional size.

7. Document the distance between tumor bed and surgical resection margins in the gross description.

8. Estimate percentage of gross necrosis that will be correlated with the estimated necrosis on the microscopic slides.

**Sampling**

How to sample the suitable area for assessment of response to neoadjuvant therapy

1. Following the tumor bed measurement, the surgical specimens may be processed fresh or with routinefixation in 10% neutral buffered formalin for at least 6 hours and no longer than 48 hours.

2. Cases with marked necrosis and cavitation are difficult to cut fresh. Overnightfixation may be helpful in such cases

3. If the tumor is small (?3 cm), it should be entirely sampled.

4. If the tumor is larger than 3 cm, an approximately 0.5 cm thick cross-section of tumor in its maximum dimension should be made and photographed.

5. On this gross photograph, a map of the complete histologic sectioning corresponding to the submitted blocks should be superimposed (See Fig. 5). These photographs should be saved with the pathologic report, electronically if possible. Additional histologic sections can be submitted if desired.

6. Histologic sections at the periphery of the tumor should include the border of the tumor with at least 1 cm of the surrounding nonneoplastic lung parenchyma to define the edge of the tumor.

**Histologic Assessment of Primary Tumors**

How to define the border of the tumor bed from surrounding non-neoplastic lung

1. Identify reactive changes in surrounding non-neoplastic lung (for example: organizing pneumonia, interstitial fibrosis, hemorrhage, marked type II pneumocyte hyperplasia or reactive atypia, and inflammatory infiltrates).

2. Inflammatory cells that are part of the reactive changes surrounding the tumor bed must be distinguished from tumor stromal inflammation where the inflammatory cells should be confined to the tumor bed.

3. The true tumor bed should consist only of viable tumor along with concurrent necrosis and stroma which includes both fibrosis and inflammation. The size of tumor bed should be adjusted for histologic changes related to neoadjuvant treatment in the surrounding lung.

4. Correlate with the gross photograph with mapping of histologic sections to determine whether the gross measurement of the tumor bed size is an accurate assessment or if it includes non-neoplastic reactive changes.

How to record the histologic features in tumor bed

1. The percentages of viable tumor, stromal tissue, and necrosis should be estimated on the basis of the review of the microscopic sections on each slide and then the total percentage of viable tumor is estimated. The percentage should total 100% of the tumor bed.

2. Each component should be assessed in 10% increments unless the amount is less than 5% when an estimate of single percentages should be recorded.

3. There are two stromal tissue components: fibrosis and inflammation. Although more detailed assessment of stroma can be made (Fibrosis: dense hyalinized connective tissue, fibroelastotic scarring, and loose or myxoid connective tissue. Inflammation: chronic inflammation, acute inflammation, histiocytes, xanthogranulomatous, cholesterol clefts, and granulomatous reaction), until there is sufficient validation that any of these are clinically relevant, recording of these features is not needed for routine clinical purposes.

**Determination of the Pathologic Response to Neoadjuvant Therapy**

1) The final pathologic response should be determined on the basis of the histologic features correlated with the gross findings. Particularly the mapped gross photograph and corresponding histologic sections can be helpful, especially in markedly necrotic and cavitated tumors.

2) Until digital and, or computational approaches are routinely available, a semiquantitative approach can be done.

^a^ For processing of lymph nodes, see recommendation 9.

**Recommended Synoptic Template for Recording Lung Cancers After Neoadjuvant Therapy**

**Primary Tumor**

Type of neoadjuvant therapy

a. No known presurgical therapy

b. Type of neoadjuvant therapy:

a. Chemotherapy ____

b. Radiotherapy ____

c. Immunotherapy (Please specify) _________

d. TKI (please specify) ________

e. Other (please specify) ________

Treatment effect in primary tumor

a. Percentage of viable tumor: ___% (record in 10% increments except below 10%, then record single digits between 1%–5%)a

b. No residual viable tumor identified

c. Percentage of necrosis: ____%

d. Percentage of stroma (includesfibrosis and inflammation): ____%

Grade of inflammation (choose the appropriate grade)

___ Mild

___ Moderate

___ Marked

Method (choose all what was used for evaluation)

____Correlation was made with a gross photograph of tumor cut surface: Yes ___ No ____

____ Evaluation was aided by use of tumor mapping to match a gross photograph to histologic sections: Yes ____ No ____

____Evaluation was aided by radiologic pathologic correlation: Yes ___ No ____

**Treatment Effect in Lymph Node Metastases**

a. Total number of lymph node stations examined: ____

b. Total number of lymph nodes examined: ____

c. No carcinoma present: ____

d. Total number of lymph nodes with metastatic carcinoma: ____

e. Lymph node stations involved by tumor with treatment related changes: ______

f. Lymph node stations with treatment-related changes without viable tumor: _______

g. Largest tumor focus: _____mm at station number: ____

h. Extracapsular extension present: ______

i. No extracapsular extension: _______

Comment: _________________________________

^a^ The three components: % viable tumor, % necrosis, and % stroma should add up to 100%.

**Abbreviations**

| AE | Adverse event |
| --- | --- |
| ANC | Absolute neutrophil count |
| CFDA | China Food and Drug Administration |
| CR | Complete response |
| CTCAE | Common Terminology Criteria for Adverse Events |
| CTLA-4 | Cytotoxic T lymphocyte antigen-4 |
| DCR | Disease control rate |
| DLT | Dose-limiting toxicity |
| DFS | Disease-free survival |
| ECG | Electrocardiogram |
| ECOG PS | Eastern Cooperative Oncology Group Performance Status |
| eCRF | Electronic Case Report Form |
| FBG | Fasting blood glucose |
| HB | Hemoglobin |
| HBV | Hepatitis B virus |
| HCT | Hematocrit |
| HCV | Hepatitis C |
| HIV | Human immunodeficiency virus |
| ICF | Informed consent form |
| ICIs | Immune checkpoint inhibitors |
| IgG4 | Immunoglobulin G4 |
| irAE | Immune-related adverse event |
| MedDRA | Medical Dictionary for Regulatory Activities |
| MPR | Major pathological response |
| NCI | National Cancer Institute |
| NSCLC | Non-small-cell lung cancer |
| NYHA | New York Heart Association |
| ORR | Objective response rate |
| OS | Overall survival |
| PD | Progressive disease |
| PD‑1 | Programmed death 1 |
| PD‑L1 | Programmed death-ligand 1 |
| PFS | Progression-free survival |
| pCR | Pathologic complete response |
| PLT | Platelet count |
| PR | Partial response |
| Q2W | every 2 weeks |
| Q3W | every 3 weeks |
| RECIST | Response Evaluation Criteria in Solid Tumors |
| SAE | Serious adverse event |
| SD | Stable disease |
| SCLC | Small cell lung cancer |
| sCr | Serum creatinine |
| TMB | Tumor mutational burden |
| TBIL | Total bilirubin |
| TSH | Thyroid-stimulating hormone |
| TSH | Thyroid-stimulating hormone |

**Signature Page**

**Study drug:** Sintilimab (R & D code: IBI308)

**Trial Name:** Neoadjuvant of Sintilimab combined with Chemotherapy for Resectable NSCLC（neoSCORE）：A Prospective, Randomized, Open-Label, Single-Center Phase 2 Trial

**Protocol No.:** 2020-KYY-518052-0092

**Principal investigator (signature):**  **Date:**  1
